# The Role of Infant Health Problems in Constraining Interneighborhood Mobility: Implications for Citywide Employment Networks

**DOI:** 10.1177/00221465231172176

**Published:** 2023-06-04

**Authors:** Megan Evans, Corina Graif, Stephen A. Matthews

**Affiliations:** 1 Pennsylvania State University, University Park, PA, USA

**Keywords:** commuting networks, infant health, neighborhood inequality, social isolation, spatial mobility

## Abstract

Infant health problems are a persistent concern across the United States, disproportionally affecting socioeconomically vulnerable communities. We investigate how inequalities in infant health contribute to differences in interneighborhood commuting mobility and shape neighborhoods’ embeddedness in the citywide structure of employment networks in Chicago over a 14-year period. We use the Census Bureau’s Longitudinal Employer–Household Dynamics’ Origin–Destination Employment Statistics to analyze commuting networks between 2002 and 2015. Results from longitudinal network analyses indicate two main patterns. First, after the Great Recession, a community’s infant health problems began to significantly predict isolation from the citywide employment network. Second, pairwise dissimilarity in infant health problems predicts a lower likelihood of mobility ties between communities throughout the entire study period. The findings suggest that infant health problems present a fundamental barrier for communities in equally accessing the full range of jobs and opportunities across the city—compounding existing inequalities.

Despite tremendous resources and ranking in the top 10 of gross domestic product, the United States ranks 33rd out of 36 OECD (2022) countries in infant mortality, below countries like the Slovak Republic and Lithuania. About 1 in 10 infants born in the United States in 2019 were born preterm (CDC 2020)—an important statistic given that infants born preterm have higher rates of death and disability, including problems related to breathing, feeding, hearing, vision, cerebral palsy, and developmental delays.^1^ However, poor infant health outcomes are not uniformly distributed across the country, and many communities fare much worse than the national average. For example, in 2017, Chicago’s infant mortality rate was 6.6 (per 1,000 live births; City of Chicago 2019), compared to a national rate of 5.8 (CDC 2020). Ever since the Chicago School, research has historically shown that infant health problems are unequally distributed across space and population groups (Blumenshine et al. 2010; Shaw and McKay 1942; Siddiqi et al. 2016). For instance, in the United States, Black women have preterm births at a rate 50% higher than Hispanic and non-Hispanic White women, and the Black infant mortality rate is more than double that of Hispanics and non-Hispanic Whites (10.8 deaths per 1,000 live births compared to 4.9 and 4.6, respectively, in 2018; CDC 2020). Place-based differences in infant health problems are known to emerge as a result of racial segregation and socioeconomic vulnerabilities (Braveman, Egerter, and Mockenhaupt 2011; Culhane and Elo 2005; Gorman 1999; Reidpath and Allotey 2003; Williams 2002).

Infant health problems deepen existing inequalities. Prior research has underscored the value of infant health as a key indicator of the broader health and social well-being of a community’s population (Alexander and Root 2022; Conley and Springer 2001), with long-term implications for population health and inequality (Torche and Rauf 2021). Family health issues have been shown to lower individual and family housing mobility (Arcaya et al. 2015), work productivity (Alavinia, Molenaar, and Burdorf 2009), and job loss (Kessler et al. 2001). Yet the extent to which infant health problems further shape place-based inequalities is less understood. It may be that the problems of residentially segregated communities are exacerbated when large groups of people burdened by infant health problems become less able to move freely across space or connect to the full range of places, opportunities, and jobs across the city. This study aims to bridge this gap by examining whether inequalities in infant health deepen existing geographic disparities by decreasing population groups’ spatial mobility and increasing entire communities’ isolation from citywide employment networks. It aims to advance existing knowledge on urban mobility and health by investigating the extent to which preterm births, low birth weight, and infant mortality combined impact population mobility patterns and influence inequality in commuting ties across urban neighborhoods in a large U.S. city.

Understanding how infant health problems shape employment-related mobility among large population groups and shape interneighborhood^2^ connections to citywide networks is an important and urgent need. A growing body of literature has shown that neighborhoods connect to one another within the city through residents’ housing mobility (Sampson and Sharkey 2008), co-offending partnerships (Papachristos and Bastomski 2018; Schaefer 2012), commuting (Graif, Lungeanu, and Yetter 2017; Kelling et al. 2021; Newmyer, Evans, and Graif 2022), and daily activity patterns (Browning, Calder, Boettner, and Smith 2017; Browning, Calder, Soller et al. 2017; Browning, Soller, and Jackson 2015; Levy, Phillips, and Sampson 2020). Despite the wide range in the types of spatial mobility and connectivity explored, these studies indicate that external connections are just as important for neighborhood outcomes as what occurs internally. Access to external resources and ties to influential political actors and organizations can lead to investments or disinvestments in the community (Bursik and Grasmick 1999; Hunter 1985; Logan and Molotch 1987) and facilitate the transfer of information, resources, norms, and social support that may not be accessible otherwise (Carpiano 2006; Lyons, Vélez, and Santoro 2013).

However, limited research has examined the forces that shape neighborhoods’ extralocal connections. While past research has highlighted the spatial mismatch between where people live and work, focusing on the burden of commuting times and transportation challenges (Gobillon, Selod, and Zenou 2007; Kain 1968, 2004), much less is known about the specific neighborhoods to which people commute and about how such connectivity between neighborhoods is associated with inequalities in health above and beyond existing segregation patterns. We focus on interconnections based on commuting flows because commuting is routine, usually a day-to-day activity for adults. Beyond the home, the second most common anchor of spatial activity for adults is the workplace (Kahneman et al. 2004). Of all environments other than home communities that are likely to influence people’s behaviors and outcomes, the work neighborhoods have the potential to be among the most influential.

Thus, this study aims to address several important gaps in the literature. First, in contrast to studies that primarily focus on individuals affected by infant health problems, this Chicago-based ecological study investigates the impact of infant health problems on a community as a whole. Second, in contrast to existing studies that focus on communities as independent units or connected through geographic contiguity, we conceptualize communities as connected through broader social ties within the larger structure of citywide exchanges of resources, information, norms, and influence. We draw on Browning, Calder, Boettner, and Smith (2017) and Browning, Calder, Soller et al.’s (2017) concept of *econetworks*—ecological networks that link residents and communities to each other because of overlapping spaces of routine activities. We focus on commuting in particular, given the fact that commuting behaviors typically represent key routine activity and daily mobility flows between communities.

We explore the extent to which the recurrent ebb and flow of commuting behaviors create a large network structure that connects the communities in which people live and work. This approach allows for a unique understanding of the relational connections between communities of people while also accounting for the larger network structure of the entire city. Unlike standard models that require independent observations, we use network models to analyze observations that are not independent of each other and account for structural controls, such as features of the entire network, including density or reciprocity of ties. This study, to our knowledge, is the first adoption of a dynamic network analysis method (temporal exponential random graph models [TERGMs]) in examining linkages between population health and mobility flows across an entire city.

## Background

### Infant Health Inequality and Isolation from Citywide Employment

An emerging literature has started to highlight the role of child health as an important selection factor in constraining the residential mobility of individuals across space (e.g., Arcaya et al. 2015). In this study, we draw on this work and extend it to examine how infant health at the community level is associated with selection processes shaping job-related mobility among communities and connectivity to the citywide employment network. Where the ecological research on infant health is less abundant, we draw on insights from existing evidence on individuals and organizations.

We draw on the precedence in the sociological and demographic literature of infant health as a key social indicator of a population’s overall health and well-being (Alexander and Root 2022; Conley and Springer 2001; Morris 1979). The health of infants has been studied not only as a community outcome on its own (Conley and Springer 2001; Light and Marshall 2018; Torche and Rauf 2021; Wildeman 2012) but, importantly, also as an explanatory indicator of the obstacles to community social and economic development, leading to inequalities across the world (Bütikofer, Løken, and Salvanes 2019; Gonzalez and Gilleskie 2017; Kalemli-Ozcan 2002; Rocco et al. 2021; Soares 2005). Birth outcomes and infant health issues have been linked to future developmental problems and morbidity and to differences in community-level educational and economic outcomes (Alderman and Behrman 2006; Behrman and Rosenzweig 2004; Conley, Strully, and Bennett 2003). Infant health and birth outcomes also stand out as particularly responsive to variations in environmental stressors at smaller timescales than other health indicators (Dunkel Schetter 2011; Torche 2011). The putative sensitivity of infant health to changes in community well-being over short periods of time makes it a particularly meaningful measure in longitudinal studies. Indeed, Conley and Springer (2001) even suggest that infant health can be considered a heuristic for community health and social welfare.

Caring for infants in general but especially for preterm or low weight infants, dealing with a child’s health complications, or losing an infant can be tremendously taxing physically and emotionally (Dyregrov and Matthiesen 1991; George et al. 2007), with implications for parents’ own health and work absences. Extensive evidence shows that infant health problems come with significant financial, emotional, and temporal burdens on families. Cuevas et al. (2005) found that preterm and low birth weight infants incurred significantly higher hospital charges and frequency of acute care visits and rehospitalizations than normal weight and full-term infants. Similarly, Petrou et al. (2003) found that preterm infants born before 32 weeks gestation spend 8 times more days in the hospital during the first 5 years of life compared to full-term infants, and the number doubled even further when duration of life was accounted for. The cost of care for very low birth weight infants (under 1,000 g) during the first 2 years of life was 25 times higher than that of normal weight infants (Tommiska, Tuominen, and Fellman 2003).

Health issues have been shown to affect productivity and work cutbacks (Alavinia et al. 2009; Kessler et al. 2001). Moreover, preterm births have long-term consequences for children (Moster, Lie, and Markestad 2008), which can make it harder for parents to find or keep a job with longer commuting needs. Studies have found substantial transportation burdens for parents of very low birth infants (McCormick et al. 1991) and personal costs, including time burdens among poor women with high-risk prenatal care attendance (Stringer 1998). Furthermore, commuting and especially commuting for long distances or through heavy traffic have been associated with negative affect (Kahneman et al. 2004), stress, and health problems (Gee and Takeuchi 2004), all of which can influence people’s job prospects, especially for married women and low-income population groups (Roberts, Hodgson, and Dolan 2011). Members of households that experience stress and time constraints related to infant health are more vulnerable and likely to cut long commuting times compared to families who do not have the additional burden of a sick infant.

Communities that experience more health problems such as high rates of infant mortality, preterm births, or low or very low birth weight have higher proportions of parents experiencing corresponding strain. When infant health problems emerge in people’s lives or the lives of their loved ones, they can be laid off, become less desirable to potential employers, or be less able or willing to commute long distances. Communities with high levels of health problems may, as a result, have more residents who become unemployed or unable to commute long distances. These burdens affect not just families’ well-being, employment, and income levels but also influence their ability to participate in community life, help neighbors, and share job-related information and resources. Thus, the more families with infant health problems there are in a community, the more limited time and opportunities for interactions in the community and the more likely community life and social capital will suffer as a whole (Carpiano 2006; Kawachi et al. 1997; Kawachi, Subramanian, and Kim 2008; Putnam 2000).

Indeed, when many residents experience health problems, the aggregated patterns can reverberate and multiply throughout the entire community. When fewer people stay employed because of health issues, fewer people can advise or recommend friends and neighbors in job searches and thus connect them to employment across the city (Granovetter 1973; Hellerstein, Kutzbach, and Neumark 2014; Hellerstein, McInerney, and Neumark 2011). Evidence exists that people often rely on neighbors and acquaintances for job search connections (Granovetter 1973; Lin and Dumin 1986; Marin 2012). Neighbors provide information about job openings in general and the employers who eventually hire them (Hellerstein et al. 2014), especially in the case of lower skill jobs (Hellerstein et al. 2011). If residents looking for new jobs have a more limited pool of information accessible through their neighbors overall, the social mobility of residents and the social capital of the community overall will be diminished. In sum, we expect that:

*Health Isolation Hypothesis*: Higher levels of infant health problems in a community decrease the chances of commuting ties to other communities in Chicago

### Homophily in the Citywide Employment Network

The social network concept of homophily, colloquially known as “birds of a feather flock together” (McPherson, Smith-Lovin, and Cook 2001), represents the idea that people are more likely to share information and resources with others if they share characteristics like race, ethnicity, education, and gender. A large literature in social networks has described such socially homophilous effects, which extend beyond ties between people to ties between organizations and communities (Ibarra 1995; Marsden 1988; McPherson et al. 2001; Neal 2013). Especially under difficult circumstances, similarity in background has been shown to activate connections between organizations (Galaskiewicz and Shatin 1981). On the flip side, dissimilarity may also contribute to exclusion—preventing or dissolving connections. Growing examples of connections between communities exhibiting ecological homophily patterns (Graif, Gladfelter, and Matthews 2014) are found in studies of community leadership (Sampson 2012), residential mobility flows across space (Sampson and Sharkey 2008), commuting (Graif et al. 2017), and even co-offending (Schaefer 2012). While we still understand little about how such homophilous patterns are generated, the ubiquitous nature of work relationships across urban areas may mean that job connections are likely related to many of these already discovered ties between communities.

In communities where infant health problems are common, employers who formally accommodate employees’ needs can help prevent absenteeism and turnover and save money (Halpern 2005; Lourel et al. 2009). Informal flexible arrangements, norms, or support systems can also emerge to cover for coworkers who need to take days off for a sick child (De Menezes and Kelliher 2017; Holt and Thaulow 1996). Immediate coworkers and supervisors can help with work flexibility and provide informal buffers in otherwise unsupportive organizational cultures (Blair-Loy and Wharton 2002). Previous experience with health issues could influence some employers and coworkers to offer emotional and social support (Sloan, Evenson Newhouse, and Thompson 2013) or advice and information regarding health insurance, doctors, time management, childcare, and social support. Such support would increase the chances that workers will maintain existing commuting ties. Importantly, flexible arrangements and support systems help more employees than just those with infant health problems. At the ecological level, this would contribute to stability and growth in commuting between communities with similar health issues.

Conversely, a mismatch in health status between residential communities and work communities may be associated with a mismatch in expectations around workers’ absences and norms in understanding around the need for flexible work arrangements (Grice, McGovern, and Alexander 2008). Such mismatch could render existing heterophilous ties, between communities dissimilar in health, more vulnerable to dissolution and prospective new ties (e.g., new hires) less likely to form. In sum, we expect that:

*Health Inequality Hypothesis*: The more dissimilar in infant health two communities are, the lower the chances of a commuting tie to exist between them.

### Heterogeneity in Health Effects Due to Recession Disruptions

Closely related to the two main hypotheses, we are also interested in understanding the extent to which health effects would vary because of network disruptions due to the Great Recession. Like employment, commuting ties are inevitably sensitive to disruptions during economic downturns (Kim and Horner 2021). During the Great Recession of 2008, millions of people lost their jobs because many employers closed their doors or relocated to other states or countries (Grusky, Western, and Wimer 2011; Kalleberg and von Wacher 2017). According to the U.S. Bureau of Labor Statistics (2010), the unemployment rate in the Chicago metro area more than doubled, from 3.6 in 2006 (and 4.9 in 2007) to 10.0 in 2009. The Great Recession’s link to health is complex at the community level, yet evidence suggests it created a myriad of hardships for individuals, including health insurance losses and increases in material, physical, and mental strain (Burgard and Kalousova 2015; Burgard and Lin 2013). Thus, we examine the extent to which the Great Recession amplified the negative effects of infant health problems on job disconnectedness and econetwork inequality.

## Data And Methods

### Data

This study investigated infant health and employment networks in Chicago. While Chicago is a highly segregated city, it sees racial disparities in infant health problems also comparable with national rates, with a Black infant health mortality rate 3 times higher than White infants and 2 times higher than Hispanic infants. Studies show that spatial processes operating within Chicago’s neighborhoods are similar to those happening in other large metropolitan cities globally (Sampson 2012).

Data for this study came from the Longitudinal Employer–Household Dynamics (LEHD) Origin–Destination Employment Statistics (LODES; Abowd and Vilhuber 2011; Graham, Kutzbach, and McKenzie 2014), decennial census (U.S. Census Bureau 2000), American Community Survey (ACS; U.S. Census Bureau 2012), and the City of Chicago’s (2019) Data Portal. The LEHD is a program sponsored by the U.S. census with the aim to represent workforce dynamics. By combining unemployment insurance earning records with administrative and survey data on firms, workers, and households, LEHD calculates commuting flow statistics. While these data exclude federal employees before 2010 (about 2% of workers), it contains employee data on nonprofit and religious institutions, informal workers, and the self-employed (Graham et al. 2014). Hence, these data presented us with comprehensive commuting flow statistics connecting employer/establishment location to employees’ home locations. We structured these data to create a full interneighborhood commuting network. Focusing on the city of Chicago, we generated an interneighborhood commuting network among Chicago’s 77 Community Areas^3^ for 14 consecutive years (2002–2015).

We used the City of Chicago’s Data Portal, which combines data from multiple institutions and aggregates it to the Community Area level. It includes data provided by the Illinois Department of Public Health on infant mortality, low birth weight, very low birth weight, and preterm births (City of Chicago 2019). These data are released in five-year aggregations for 2000 to 2004, 2005 to 2009, 2010 to 2014, 2011 to 2015, 2012 to 2016, and 2013 to 2017. The U.S. Census Bureau’s 2000 decennial census and 2008 to 2012 five-year estimate ACS data provided us with the sociodemographic information of our Community Areas.

### Dependent Variable

Our dependent variable was a commuting tie between two communities. We calculated this relation using the LEHD’s LODES data on the number of persons from each home community commuting to each work community. This created a 77 by 77 valued matrix representing each of Chicago’s Community Area commuting relations. We normalized these values to the 2000 population level of the home community so that we could define a commuting tie between two communities if at least .5% of the home community’s residents commute to the work community. We did this by dichotomizing the matrix where a tie exists (=1) if the cell’s value was higher than .5% and a tie did not exist (=0) if the cell’s value was less than .5% of the community’s population. This yielded a binarized, directed commuting network of Chicago’s Community Areas. We created a unique commuting network for each year included in our study (2002–2015). On average, Community Areas in Chicago have a population of 35,000 residents, meaning that a .5% cutoff represents 175 residents commuting from a home community to a work community.^4^ This threshold yielded a large enough sample that (a) enabled us to avoid noisy data where all communities would be connected to one another and (b) represented a substantively meaningful number of residents commuting from the home to work community. We explore alternative commuting thresholds in Appendix E in the online version of the article.

### Independent Variables

To measure each community’s quality of infant health, we created a standardized, continuous indicator of infant health problems, with higher, positive numbers meaning worse health. This measure was created for each of the six five-year periods available in the data (i.e., 2000–2004, 2005–2009, 2010–2014, 2011–2015, 2012–2016, and 2013–2017). The indicator of infant health included the standardized versions of four health measures from the City of Chicago’s Data Portal at the level of the Community Area: the rate of infant mortality, the percent of preterm births, the percent of babies born with low birth weight, and the percent of babies born with very low birth weight. The internal reliability of our infant health indicator was high (range = .95–.97 for all periods).

After creating the standardized infant health indicator for each of the six available time intervals, we used linear interpolation. We assigned the 2000 to 2004 measure as the 2002 value, the 2005 to 2009 measure as the 2007 value, the 2010 to 2014 measure as the 2012 value, the 2011 to 2015 measure as the 2013 value, the 2012 to 2016 measure as the 2014 value, and the 2013 to 2017 measure as the 2015 value. Data on the remaining years (2003–2006 and 2008–2011) were interpolated.

We used our indicator of infant health to predict our commuting network in two ways. First, we examined how infant health in the home community influenced the likelihood that it would send ties out into the network, assessing its influence on network activity (home community relations). Second, we examined how the absolute difference between the home and work communities’ quality of infant health influenced the likelihood a tie would exist between community pairs (dyads), predicting network dissimilarity. While we also predicted work community relations (the likelihood a community will receive ties from others in the network, or network popularity), our main independent variables of interest were home community relations (i.e., network activity) and dissimilarity in infant health problems.

### Controls

We accounted for several community-level sociodemographic variables that influence a community’s position within the commuting network. We controlled for residential stability, population density, and a community’s majority racial composition. These measures were created using data from the 2000 decennial census and the 2008 to 2012 ACS. The 2008 to 2012 measures were assigned to the year 2010, and we used linear interpolation and extrapolation to obtain values for the missing years. We used a factor weighted principal component analysis to calculate *residential stability* using the percentage of owner-occupied housing and the percentage of residents five years old and older who resided in the same house five years earlier. We standardized the *population density* of each Community Area to a mean of 0 and a standard deviation of 1. We created three variables to capture the extent to which a community is segregated by indicating whether the community was majority Black, majority Hispanic, or majority White. These three variables were dichotomous measures indicating whether at least 60% of the community’s population is the select racial-ethnic group (=1). We chose this threshold to convey a high concentration of a group, beyond the standard majority, while also preserving a balanced distribution of areas across the categories (Graif et al. 2017). Finally, we also accounted for the commuting density of a community (i.e., the proportion of employees that commute into the Community Area for work). All of the nodal attributes included were assessed the same way that we assessed infant health problems (i.e., as work and home community relations as well as dissimilarity).

We also accounted for two relational characteristics between communities. We controlled for spatial proximity and public transportation ties. Spatial proximity was defined as spatial contiguity (Queen 1 criterion; i.e., if two communities were directly adjacent with a shared boundary or vertex, =1). We created a 77 by 77 undirected network of spatial proximity and applied it in our analysis as an edge attribute. Public transportation ties were defined as a tie between two Community Areas that shared a mass transit (either bus or rail) line. We created a 77 by 77 valued network of public transportation where a tie existed between two Community Areas if they shared a mass transit connection. The value of each transportation tie represented the number of mass transit lines that was shared between the two Community Areas. If two Community Areas did not share any mass transit connections, then a tie did not exist between them (=0).

### Analytic Strategy

We used the longitudinal extension of exponential random graph models (ERGMs Hunter et al. 2008), TERGMs (Krivitsky and Handcock 2019), to investigate which community-level attributes predicted the existence of a commuting tie in our commuting relation network of Chicago. A key assumption of typical regression models is that observations are independent. Network data violate this assumption, our case being that commuting ties between Community Areas were interdependent. The ERGM overcomes this limitation of traditional regression models by controlling for the structural effects of the data (i.e., the endogenous effects). The endogenous effects our models controlled for when predicting our commuting network were the number of edges in the network, reciprocity, the geometrically weighted indegree distribution, number of edgewise shared partners, and number of dyadic shared partners. Because we were assessing a longitudinal network with a TERGM, we also controlled for two network statistics capturing temporal effects. We controlled for whether there was a *linear time trend* characterizing the networks and whether there was stability in the persistence of ties from one year to the next using a *memory* term.

The ERGM and its longitudinal extension, the TERGM, predicts the probability of pairwise patterns in the given network (Graif et al. 2017; Hunter 2007; Leifeld, Cranmer, and Desmarais 2017; McMillan 2019). The ERGM estimates the true likelihood function and assesses if any of the network structures are more or less likely to occur than chance (Hunter 2007). Our TERGM terms can be interpreted in the same fashion as ERGM terms but present the average effect of the network statistics across the 14-year period. More detailed information on our analytic strategy can be found in Appendix A in the online version of the article.

## Results

### Visualizing Commuting Flows across Geographic Space

**Figure 1.**
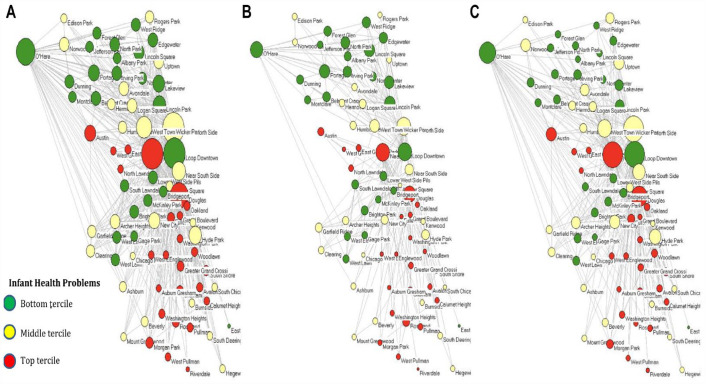
Infant Health by Tercile Clusters and Chicago’s Interneighborhood Commuting Mobility Network in 2002. (a) All Commuting Ties. (b) Only Homophilous Commuting Ties within Each of the Three Health Clusters of Nodes. (c) Only Heterophilous Commuting Ties between the Three Health Clusters of Nodes. *Note*: Nodes (*N* = 77) represent communities located in space according to the geographic coordinates of their centroid. Node size represents the degree centrality (i.e., number of commuting ties) according to the category of ties represented in each graph. Green represents communities in the bottom tercile of infant health problems, yellow represents the middle tercile, and red represents the top tercile. Commuting data come from the Longitudinal Employer–Household Dynamics Origin–Destination Employment Statistics, and infant health data come from the Illinois Department of Public Health.

**Figure 2.**
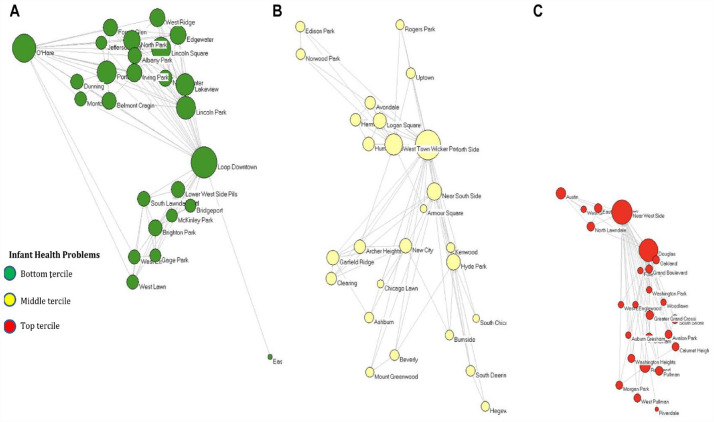
Interneighborhood Commuting Mobility Networks of Homophilous Ties by Infant Health Clusters in 2002. *Note*: Only within-cluster ties are shown, one health cluster at a time. Nodes (*N* = 77) represent communities located in space according to the geographic coordinates of their centroid. Node size represents the degree centrality (i.e., number of commuting ties) according to the category of ties represented in each graph. Green represents communities in the bottom tercile of infant health problems, yellow represents the middle tercile, and red represents the top tercile. Commuting data come from the Longitudinal Employer–Household Dynamics Origin–Destination Employment Statistics, and infant health data come from the Illinois Department of Public Health.

**Figure 3.**
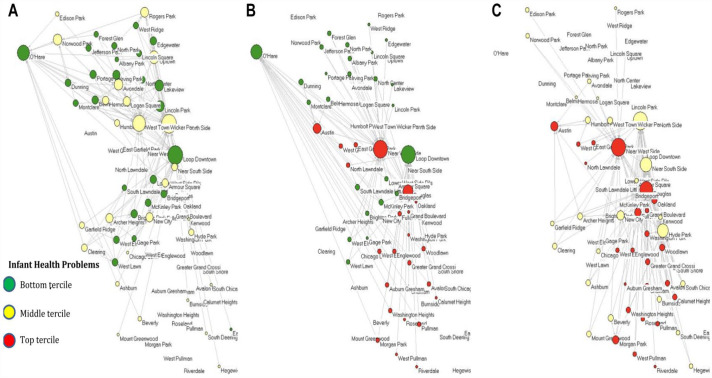
Interneighborhood Commuting Mobility Network for Heterophilous Ties between Infant Health Clusters in 2002. *Note*: Only between-clusters ties are shown, two health clusters at a time. Nodes (*N* = 77) represent communities located in space according to the geographic coordinates of their centroid. Node size represents the degree centrality (i.e., number of commuting ties) according to the category of ties represented in each graph. Green represents communities in the bottom tercile of infant health problems, yellow represents the middle tercile, and red represents the top tercile. Commuting data come from the Longitudinal Employer–Household Dynamics Origin–Destination Employment Statistics, and infant health data come from the Illinois Department of Public Health.

Figures 1 through 3 present the Chicago interneighborhood commuting network for 2002 with the nodes positioned according to the geographic coordinates of their centroids. Across all three figures, green nodes represent communities within the lowest tercile of infant health problems, red nodes are those within the highest tercile of infant health problems, while yellow communities are those in the middle of the distribution. Figure 1a presents the entire network of nodes and ties with the nodes sized by degree (i.e., the number of both outgoing and incoming commuting ties the community has). This figure highlights that communities with the highest level of infant health problems have the fewest commuting ties in the network. Figure 1b shows only the homophilous commuting ties within infant health clusters, while Figure 1c shows only heterophilous commuting ties within infant health clusters. Figures 1b and 1c are further expanded on in Figures 2 and 3 for more in-depth visualization.

Figure 2 presents only the homophilous ties within infant health clusters, with Figure 2a presenting communities within the lowest level of infant health problems, Figure 2b presenting those within the middle of the distribution, and Figure 2c presenting those with the highest level of infant health problems. Figure 3 presents only the heterophilous ties between the infant health clusters, with Figure 3a presenting heterophilous ties between the lowest and middle terciles of infant health problems, Figure 3b being those between the lowest and highest terciles, and Figure 3c being between the middle and highest terciles.

Figure 2 demonstrates that communities across the infant health problem clusters are highly interconnected. The homophilous networks highlight the bounded worlds that the nodes exist within and the level of network segregation that occurs conditioned by infant health problems. Figure 3 suggests that communities in the lowest and middle infant health problem clusters have more heterophilous ties with one another than either community has with the highest infant health problem cluster. The heterophilous networks highlight the existing avenues for potential resource and information flow across communities of differing levels of infant health problems. Such ties may represent silver linings in the ability of communities burdened by infant health problems to connect more widely to the rest of the commuting network.

Table 1 shows the descriptive statistics for the community nodes for the last year of the main measures in our analysis, including infant health problems, racial composition, sociodemographic indices, and the commuting density. In 2015, 36% of communities were majority Black, 17% were majority White, and 15% were majority Hispanic. Table 1 also presents the descriptive statistics of the commuting network, including the indegree and outdegree (the number of other communities that an area is connected to through receiving or sending ties, respectively). In 2015, the average community both sent and received seven commuting ties, although the range is much higher among receiving ties (indegree) than sending ties (outdegree). During the prerecession period, a community sent and received about one additional commuting tie than during the recession and after.

**Table 1.** Descriptive Statistics for 2015, *N* = 77.

	Mean	SD	Minimum	Maximum
Community attributes				
Infant health problems	.00	.99	–1.64	1.98
Residential stability	.00	1.14	–2.19	2.27
Population density	.00	1.14	–1.05	5.07
Commuter density	.02	1.11	–.24	9.49
Mostly Black	.36	.48	0	1
Mostly White	.17	.38	0	1
Mostly Hispanic	.16	.37	0	1
Network attributes				
Tie threshold, .5% cutoff				
Indegree	7.01	16.53	0	76
Outdegree	7.01	2.90	3	17
Time period, 0.5% cutoff				
Prerecession average				
Indegree	7.76	16.05	0	76
Outdegree	7.76	2.33	2	13
Recession and postrecession average				
Indegree	7.01	16.10	0	76
Outdegree	7.01	2.33	3	14

*Note*: Commuting data come from the Longitudinal Employer–Household Dynamics Origin–Destination Employment Statistics, infant health data come from the Illinois Department of Public Health, and sociodemographic data come from the U.S. Census Bureau.

### Infant Health and Commuting Networks

Table 2 presents the TERGM results assessing the role of infant health problems on a community's connections to others across the Chicago’s commuting network over time. The models in Table 2 present the average effects of each of the covariates on the log odds of a tie existing, conditional on the rest of the network (Leifeld et al. 2017). The first model includes controls for the network structure, relational effects, and time effects and our infant health predictors. Model 2 includes measures for the community sociodemographic composition. Our results indicate that the final model is the best fit to the data with the lowest Akaike information criterion and the best model fit statistics, which can be found in Appendix B in the online version of the article.

**Table 2.** Bootstrapped Temporal Exponential Random Graph Models Assessing Infant Health Problems and Commuting Ties, 2002 to 2015, *N* = 83,006.

Network Characteristics	Model 1				Model 2			
	Odds Ratio	Estimate		95% Confidence Interval	Odds Ratio	Estimate		95% Confidence Interval
Infant health problems								
Sender (“home” community)	1.010	.010		(–.077, .088)	.920	–.084		(–.186, .044)
Dissimilarity	.795	–.229	*	(–.336, –.101)	.704	–.351	*	(–.474, –.213)
Sender (“home” community)								
Residential stability					1.156	.145	*	(.038, .256)
Population density					.789	–.237	*	(–.365, –.094)
Commuter density					.826	–.191		(–.479, .169)
Majority Black					.856	–.156		(–.431, .073)
Majority Hispanic					.998	–.002		(–.242, .234)
Majority White					1.307	.268	*	(.101, .431)
Dissimilarity								
Residential stability					.826	–.192	*	(–.283, –.115)
Population density					.862	–.148	*	(–.212, –.068)
Commuter density					1.489	.398	*	(.029, .724)
Node match								
Majority Black					1.002	.002		(–.332, .322)
Majority Hispanic					1.537	.430	*	(.209, .677)
Majority White					1.461	.379	*	(.162, .614)
Network structure								
Edges	.134	–2.011	*	(–2.477, –1.571)	.078	–2.548	*	(–3.270, –1.896)
Mutual	.772	–.259		(–.559, .013)	.976	–.025		(–.418, .328)
Geometrical weighted indegree (popularity spread)	.089	–2.420	*	(–2.873, –1.969)	.182	–1.702	*	(–2.157, –1.234)
Geometrical weighted edgewise shared partners (closed triads)	3.477	1.246	*	(1.064, 1.420)	2.746	1.010	*	(.797, 1.209)
Geometrical weighted dyadic shared partners (open triads)	.897	–.109	*	(–.128, –.090)	.912	–.092	*	(–.110, –.074)
Multiplex relations								
Spatial proximity	1.821	.600	*	(.159, 1.018)	3.781	1.330	*	(1.010, 1.682)
Public transportation	1.163	.151	*	(.110, .206)	1.165	.153	*	(.115, .208)
Time covariates								
Linear time trend	1.017	.017		(–.029, .052)	.998	–.002		(–.045, .031)
Memory	11.051	2.402	*	(2.246, 2.609)	7.659	2.036	*	(1.884, 2.272)
Akaike information criterion				10,496.72				9,320.345

*Note*: Receiver (“work” community) coefficients for each nodal attribute are included in all models but not shown. These results can be requested from the authors. Commuting data come from the Longitudinal Employer–Household Dynamics Origin–Destination Employment Statistics, infant health data come from the Illinois Department of Public Health, and sociodemographic data come from the U.S. Census Bureau.

* *p* < .05.

Controlling for network structural properties but without any sociodemographic indices in Model 1, the analyses show that the level of infant health problems does not, at this first look, predict a community’s chances to send ties to other communities. This does not support our neighborhood health isolation hypothesis. However, the results do support our econetwork health inequality hypothesis, indicating that communities are less likely to share ties with communities who have dissimilar levels of infant health problems.

With the inclusion of the community sociodemographic indices in Model 2, we continue to find a robust association of dissimilarity in infant health problems and commuting. The magnitude of the coefficient for dissimilarity in infant health becomes stronger in Model 2 than in Model 1. Our final model finds that on average, a standard deviation difference between two communities in their level of infant health problems decreases their odds of having a commuting tie by about 30% (95% confidence interval [CI] 38%, 19%). These findings support our health inequality hypothesis, suggesting that communities are more likely to share commuting ties with communities that are similar in their level of infant health problems. Homophily is a common feature of networks (McPherson et al. 2001), and the dissimilarity coefficient indicates that homophily in infant health problems characterizes relations in the commuting network.

Model 2 also shows that communities that are spatially contiguous and share more transportation lines with each other are more likely to have commuting relations between them. Furthermore, the positively significant memory terms means that there is dyadic stability in the network (i.e., communities are likely to maintain the same commuting ties they had in the year prior). There is a negative geometrically weighted indegree coefficient, which means that popular communities in the network are not more likely to receive incoming ties just because they are popular in the network. The positively significant coefficient for closed triads and negatively significant coefficient for open triads indicates that the commuting network has a propensity for transitivity (i.e., if communities A and B share commuting ties and communities B and C share commuting ties, then communities A and C have a propensity to also share commuting ties). Additionally, the significant edge coefficient indicates that there is a lower likelihood of a commuting tie existing than at random, which is expected given that the maximum number of ties are not expected to occur in real-world networks.

Examining the sociodemographic indices in Model 2, we find a positive significant sender coefficient for majority White communities. Communities that are majority White are more likely to send commuting ties. There is also a significant coefficient for a community’s level of residential stability and population density on sending commuting ties. Higher levels of population density decrease the likelihood that communities will send commuting ties, while higher levels of residential stability increase the likelihood that communities will send commuting ties. These findings indicate that communities with higher population density will be less connected in the commuting network, while majority White communities and communities with higher residential stability will be more connected.

We find a significant homophily relation for five of the six sociodemographic indicators. Communities that are majority Hispanic and White are all more likely to send commuting ties to communities that match them in their majority racial composition. Conversely, communities more dissimilar in their racial composition will be less likely to share a tie. Higher dissimilarity between Community Areas in their residential stability and population density decreases the likelihood of a commuting tie between them. Higher dissimilarity in the commuting density, however, increases the likelihood of two communities sharing a tie. This means that communities are more likely to have commuting relations with communities that look similar in their residential stability and population density but dissimilar in their commuting density.

Overall, Model 2 does not support our health isolation hypothesis that communities with higher levels of infant health problems are less likely to send commuting ties into the employment network. However, our results do support our health inequality hypothesis that communities are more likely to share ties with communities that are similar in infant health problems.

#### The Great Recession, infant health, and commuting networks

In Table 3, we further investigate the relationship between infant health problems and isolation from the citywide employment network by examining if there is variation around the recession. We reproduce Model 2 from Table 2 by separating our longitudinal network analyses into two time periods—prerecession (2002–2006) and recession and postrecession (2007–2015).

**Table 3.** Bootstrapped Temporal Exponential Random Graph Models Assessing Prerecession (2002–2006, *n* = 29,645), Recession, and Postrecession (2007–2015, *n* = 53,361) Time Periods.

Network Characteristics	Prerecession				Recession and Postrecession			
	Odds Ratio	Estimate		95% Confidence Interval	Odds Ratio	Estimate		95% Confidence Interval
Infant health problems								
Sender (“home” community)	.937	–.065		(–.302, .307)	.885	–.122	*	(–.179, –.078)
Dissimilarity	.668	–.404	*	(–.572, –.337)	.792	–.233	*	(–.429, –.043)
Sender (“home” community)								
Residential stability	1.115	.109		(–.026, .293)	1.075	.073		(–.055, .250)
Population density	.662	–.412	*	(–.606, –.154)	.870	–.139		(–.330, .028)
Commuter density	.466	–.763	*	(–.984, –.561)	1.038	.038		(–.301, .439)
Majority Black	.952	–.049		(–.620, .198)	.758	–.278	*	(–.490, –.092)
Majority Hispanic	.941	–.061		(–.671, .370)	1.167	.154		(–.100, .421)
Majority White	1.151	.141	*	(.038, .308)	1.313	.273	*	(.013, .467)
Dissimilarity								
Residential stability	.744	–.296	*	(–.466, –.186)	.856	–.155	*	(–.267, –.066)
Population density	.845	–.169		(–.380, .090)	.857	–.154	*	(–.223, –.100)
Commuter density	2.721	1.001	*	(.639, 1.404)	1.208	.189		(–.184, .552)
Node match								
Majority Black	.837	–.178		(–.878, .276)	1.094	.090		(–.315, .542)
Majority Hispanic	1.480	.392		(–.023, .689)	1.764	.567	*	(.278, .864)
Majority White	1.191	.174		(–.272, .385)	1.570	.451	*	(.178, .771)
Network structure								
Edges	.134	–2.010	*	(–2.867, –.779)	.055	–2.900	*	(–3.743, –2.058)
Mutual	1.380	.322	*	(.237, .444)	.736	–.307		(–.872, .246)
Geometrical weighted indegree (popularity spread)	.218	–1.522	*	(–2.102, –1.218)	.185	–1.687	*	(–2.442, –.970)
Geometrical weighted edgewise shared partners (closed triads)	2.429	.887	*	(.548, 1.205)	2.702	.994	*	(.738, 1.218)
Geometrical weighted dyadic shared partners (open triads)	.902	–.104	*	(–.118, –.088)	.913	–.091	*	(–.118, –.060)
Multiplex relations								
Spatial proximity	3.181	1.157	*	(.757, 1.422)	4.568	1.519	*	(.925, 1.929)
Public transportation	1.161	.150	*	(.125, .178)	1.198	.181	*	(.117, .265)
Time covariates								
Memory	8.779	2.172	*	(2.098, 2.549)	8.219	2.106	*	(1.955, 2.398)
Akaike information criterion				2,947.083				5,337.548

*Note*: The linear time trend coefficient is not included in these models. Receiver (“work” community) coefficients for each nodal attribute are included in all models but not shown. These results can be requested from the authors. Commuting data come from the Longitudinal Employer–Household Dynamics Origin–Destination Employment Statistics, infant health data come from the Illinois Department of Public Health, and sociodemographic data come from the U.S. Census Bureau.

* *p* < .05.

These models do not find a significant sender coefficient for infant health problems in the prerecession period, consistent with the results presented in Table 2, which cover the entire period of this study. Interestingly, however, for the recession and postrecession period, we find a negative sender coefficient among communities with high infant health problems, suggesting that the health isolation hypothesis is more supported during times of economic distress. The infant health inequality hypothesis is consistently supported across both models.

Consistent across the two time periods is the positive sender coefficient for majority White communities and the homophily coefficient for residential stability. Interestingly, the role of race in a community’s connection to the citywide employment network is more pronounced in the recession and postrecession period. Net of all controls, the homophily coefficient for communities that are majority Hispanic and White found in Table 2 is only present during the recession and postrecession period. Additionally, during the recession and postrecession period, majority Black communities are less likely to send ties in the network. These results suggest that during times of economic distress, existing racial and health disparities between communities play a larger role in determining commuting flow patterns than during times of economic prosperity.

### Time-Lagged Analysis of Infant Health and Commuting Networks

In Table 4, we further investigate the relationship between infant health problems and isolation from the citywide employment network by examining whether the relationship varies across time. A time-lagged analysis assuages concerns about the directionality of the relationship between infant health and commuting and allows us to differentiate between short-term and long-term patterns of association between infant health problems and connectivity in the employment network. Table 4 reproduces Table 2, Model 2 and Table 3 using a time-lagged network where covariates measured in the year prior predict the focal year. For example, covariates from 2002 are used to predict commuting in 2003 and so forth.

**Table 4.** Bootstrapped Temporal Exponential Random Graph Models Assessing Lagged Infant Health Problems and Commuting Ties (2003–2015, *N* = 77,077), Prerecession (2003–2006, *n* = 23,716), Recession, and Postrecession (2007–2015, *n* = 53,361) Time Periods.

Network Characteristics	Entire Study Period				Prerecession				Recession and Postrecession			
	Odds Ratio	Estimate		95% Confidence Interval	Odds Ratio	Estimate		95% Confidence Interval	Odds Ratio	Estimate		95% Confidence Interval
Infant health problems												
Sender (“home” community)	.902	–.103	*	(–.191, –.006)	.928	–.074		(–.343, .277)	.884	–.123	*	(–.239, –.001)
Dissimilarity	.792	–.233	*	(–.414, –.065)	.855	–.157		(–.483, .146)	.819	–.200	*	(–.440, –.004)
Sender (“home” community)												
Residential stability	1.183	.168		(.044, .312)	1.286	.251	*	(.096, .368)	1.079	.076		(–.050, .230)
Population density	.788	–.239	*	(–.382, –.090)	.709	–.344	*	(–.728, –.104)	.854	–.158		(–.356, .016)
Commuter density	.828	–.188		(–.429, .111)	.514	–.665	*	(–.761, –.483)	.884	–.123		(–.388, .379)
Majority Black	.893	–.113		(–.306, .024)	1.052	.050		(–.220, .390)	.751	–.286	*	(–.507, –.058)
Majority Hispanic	.997	–.003		(–.234, .248)	.847	–.166		(–1.048, .417)	1.179	.164		(–.090, .440)
Majority White	1.223	.201	*	(.035, .397)	1.095	.091	*	(.060, .287)	1.288	.253		(–.005, .467)
Dissimilarity												
Residential stability	.773	–.258	*	(–.365, –.161)	.621	–.477	*	(–.623, –.320)	.822	–.196	*	(–.310, –.093)
Population density	.951	–.050		(–.129, .016)	1.059	.057		(–.334, .224)	.885	–.122	*	(–.194, –.072)
Commuter density	1.502	.407	*	(.076, .711)	2.563	.941	*	(.645, 1.082)	1.449	.371		(–.104, .652)
Node match												
Majority Black	1.126	.119		(–.153, .368)	1.066	.064		(–.281, .341)	1.099	.094		(–.321, .558)
Majority Hispanic	1.558	.444	*	(.221, .687)	1.589	.463	*	(.019, .662)	1.758	.564	*	(.269, .858)
Majority White	1.358	.306	*	(.104, .560)	1.015	.015		(–.119, .144)	1.517	.417	*	(.145, .758)
Network structure												
Edges	.075	–2.584	*	(–3.308, –1.849)	.090	–2.412	*	(–3.343, –.514)	.063	–2.765	*	(–3.606, –1.931)
Mutual	.900	–.106		(–.509, .254)	1.256	.228	*	(.007, .450)	.734	–.310		(–.927, .237)
Geometrical weighted indegree (popularity spread)	.138	–1.979	*	(–2.447, –1.381)	.162	–1.818	*	(–2.403, –1.443)	.156	–1.859	*	(–2.596, –1.094)
Geometrical weighted edgewise shared partners (closed triads)	3.026	1.107	*	(.879, 1.305)	2.822	1.037	*	(.673, 1.311)	2.849	1.047	*	(.788, 1.280)
Geometrical weighted dyadic shared partners (open triads)	.909	–.096	*	(–.113, –.078)	.901	–.105	*	(–.117, –.088)	.909	–.095	*	(–.119, –.065)
Multiplex relations												
Spatial proximity	3.487	1.249	*	(.877, 1.621)	2.794	1.027	*	(.520, 1.494)	4.353	1.471	*	(.949, 1.856)
Public transportation	1.162	.150	*	(.105, .212)	1.167	.154	*	(.127, .179)	1.188	.172	*	(.101, .258)
Time covariates												
Linear time trend	.992	–.008		(–.058, .041)								
Memory	7.490	2.014	*	(1.832, 2.285)	9.153	2.214	*	(2.214, 2.575)	8.145	2.097	*	(1.931, 2.391)
Akaike information criterion				8,886.098				2,295.004				5,452.007

*Note*: The linear time trend coefficient is not included in the recession models. Receiver (“work” community) coefficients for each nodal attribute are included in all models but not shown. These results can be requested from the authors. Commuting data come from the Longitudinal Employer–Household Dynamics Origin–Destination Employment Statistics, infant health data come from the Illinois Department of Public Health, and sociodemographic data come from the U.S. Census Bureau.

* *p* < .05.

The results indicate there is a strong dissimilarity and sender relationship between infant health and commuting during and after the recession, also reflected in the analysis investigating the entire study period. A 1 SD increase in infant health problems decreases the odds a community sends a commuting tie into the employment network by about 12% during and after the recession and by about 10% throughout the entire study period. These results support our health isolation hypothesis that more infant health problems decrease a community’s connectivity in the employment network. In the prerecession period, there is no significant long-term association between infant health problems and commuting. Still, the time-lagged analysis finds a long-term association between pairwise dissimilarity in infant health and commuting ties especially during and after the recession, consistent with the short-term models.

### Supplementary Analyses

The goodness-of-fit statistics for the final TERGM model presented in Table 2, Model 2 are shown in Appendix B in the online version of the article. We find that the simulated graphs based on our predicted TERGM model match features of our observed graph that are not explicitly modeled, indicating good model fit.

In Appendix C in the online version of the article, we present additional models assessing whether omitted variable bias explains the relationship between infant health problems and commuting ties. We investigate variables related to community socioeconomic status—unemployment rate, median household income, and residents’ education; community health status—age-adjusted total fertility rate, teen birth rate, an index of adult mortality, years of potential life loss (YPLL), and violent crime rate (homicide, assault, battery, sexual assault, domestic violence, and robbery); and community capacity for employment—density of local workers, commercial to residential zoning ratio, worker to resident ratio, total working age population, and female composition of the working age population. Dissimilarity in infant health problems remains a robust predictor of the commuting network across all alternative model specifications. The results also indicate that dissimilarity in the density of local workers, commercial to residential zoning ratio, worker to resident ratio, female composition of the working age composition, birth rate, teenage birth rate, and high school education influence the likelihood of a commuting tie between two communities.

We also assess the sensitivity of the findings to different coding decisions. In Appendix D in the online version of the article we reproduce Model 2 from Table 2 with a longitudinal network of three time points using nonoverlapping 5-year aggregations of infant health data (2000–2004, 2005–2009, and 2010–2014). We respectively assign each of these measures to commuting data for 2002, 2007, and 2012. The results are consistent with previous analyses.

In Appendix E in the online version of the article, we present results assessing different population thresholds to create commuting networks. First, we provide more descriptive statistics on using weaker and stronger global thresholds. Second, we investigate the use of a local threshold with the disparity filter specified to an α significance level of *p* < .05. Overall, our descriptive statistics indicate that the network using a .5% global cutoff matches closest the features of the local threshold network. Additionally, when using a local threshold, dissimilarity in infant health remains an important predictor of the commuting network.

Finally, in Appendix F in the online version of the article, we investigate the extent to which commuting ties predict higher or lower levels of dissimilarity in infant health. The findings indicate that once we control for community characteristics, commuting ties do not significantly predict infant health dissimilarity, suggesting that reverse causality is not a strong issue in our data.

## Discussion

This study examines the extent to which infant health problems influence job-related population mobility between Community Areas in Chicago over a period of 14 years. It investigates the extent to which inequality in infant health problems explains differences in neighborhoods’ connectivity to or isolation from the citywide network of employment. First, we find that during and after the Great Recession, a home community’s infant health problems significantly decrease the likelihood the community sends commuting ties to other communities. With time-lagged analyses, we also find that infant health problems reduce a community’s connectivity within the employment network one year later. We also observe a significant sender relationship of infant health problems in the weaker commuting tie network. These patterns, overall, support the neighborhood health isolation expectations with respect to weaker ties and to regular ties in the long run and short run under conditions of high economic strain. The results indicate that the recession further amplified the physical and emotional toll of infant health problems on individuals (Dyregrov and Matthiesen 1991; George et al. 2007) into a collective, community problem. Communities with a higher proportion of infant health problems were disconnected from the citywide employment network, reducing their ability to access resources and opportunities across the city. The recession likely made it hard for residents to deal with health issues in the family and to find or keep jobs (Burgard and Kalousova 2015; Burgard and Lin 2013; Grusky et al. 2011; Kalleberg and von Wacher 2017). Such hardships can spill over to family, friends, and neighbors, consistent with existing evidence on the role of neighbors and acquaintances in job search connections and hiring (Granovetter 1973; Hellerstein et al. 2014; Lin and Dumin 1986; Marin 2012).

Second, we find that pairwise dissimilarity in infant health is significantly associated with decreased odds of commuting ties between any two communities independent of socioeconomic and demographic controls. These findings are robust across different model specifications, including different controls and in both the prerecession and recession and postrecession periods. They also emerge in the stronger commuting tie network and in the time-lagged analysis. This pattern overall supports the health inequality hypothesis—that communities dissimilar in their infant health problems are less likely to share commuting ties with one another. These findings are consistent with ideas that work communities with a history of infant health problems may develop supportive normative climates, social support systems, and flexible, family-friendly arrangements to prevent absenteeism and turnover (Halpern 2005). Such arrangements would enable both local and nonlocal employees to navigate stressful periods caused by infant health problems and increase their abilities to keep their jobs. Indeed, evidence exists that employers have used formal accommodations in the past to prevent turnover (Lourel et al. 2009) and that informal understandings emerge at work to enable workers to deal with infant health issues (De Menezes and Kelliher 2017; Holt and Thaulow 1996; Sloan et al. 2013). The existence of formal or informal employee accommodations may additionally prompt employees to share job recommendations with family and friends in their home neighborhood (Hellerstein et al. 2014; Marin 2012), further deepening ties between communities with similar levels of infant health problems. Systematic research is needed to further explore and test such underlying processes.

It is informative to also note that an exception to the overall pattern emerges in the weaker commuting tie network, where the health inequality relationship subsides even as the health isolation relationship becomes significant instead. Additionally, in the time-lagged analysis, dissimilarity in infant health does not predict commuting ties one year later during the prerecession period. The dilution of the inequality relation in the weaker tie network has important policy implications as we consider further capitalizing on the ability of weaker ties to connect health disadvantaged communities to more advantaged areas across the city.

The current study also has several important limitations. First, we examine only one large city. The large and complex (77 × 77 origin-destination dyads over 14 years) nature of our data set contributes to correspondingly large computational costs that make it prohibitive to examine multiple cities or a larger number of neighborhoods. Data availability constrains this study to only Chicago communities. Still, Chicago’s long-standing neighborhood boundaries and wide range of relevant and available data made this case study possible. Scholars such as Koval et al. (2006:6–7) recognize that “Chicago is still the paradigmatic city for urban studies.” Our work builds off the large number of studies that use Chicago as a case study to offer insight into neighborhood processes and population health patterns (e.g., Morenoff 2003). However, future studies comparing Chicago with other cities would be valuable.^5^ Further research would also benefit from investigating the implications of strong or weak commuting ties and the processes that influence tie formation. Lastly, our study is not a causal analysis, which limits our ability to make causal interpretations. Still, we include a wide range of conservative ecological controls and examine patterns longitudinally, in contrast to standard analyses that focus on individuals only or on communities cross-sectionally.

### Contributions and Implications

The study contributes to theoretical development in medical sociology by highlighting for the first time two key macro-level mechanisms, network isolation and unequal connectivity, through which community infant health issues can contribute to new structural disadvantages for the community. First, the findings suggest that infant health problems may amplify a community’s isolation from other communities and from the broader citywide labor market. Second, we found that infant health issues deepen inequalities in communities’ econetwork location and connectivity, thereby blocking large segments of the city population from equal access to structural opportunities and resources.

Specifically, the current study first found that, especially after the Great Recession, infant health problems were significantly associated with a lower likelihood of sending commuting ties to other communities. They suggest that in the long run and in the short run, during times of deep socioeconomic distress, infant health problems contribute to substantive cuts in a community’s connectivity and access to external jobs. Connectivity can function as a central form of community social capital (Putnam 2000; Sampson and Graif 2009) because residents commuting to work on a day-to-day basis can access services, people, clubs, and information in their work community that may not be available in their residential community (Newmyer et al. 2022).

Thus, building on the medical sociology literature that has touched on the associations between health problems and social capital at the individual level (Carpiano 2006; Kawachi et al. 1997, 2008), the implications of our study’s findings highlight for the first time unique consequences of infant health issues for diminishing social capital at the community level. These results advance theoretical development in medical sociology by adding important new evidence to a growing body of work underscoring the need to understand infant health issues as a collective problem that impacts communities beyond effects on single individuals or families. This extends existing work pointing to infant health as an indicator of community well-being (Bütikofer et al. 2019; Conley and Springer 2001; Gonzalez and Gilleskie 2017; Kalemli-Ozcan 2002; Rocco et al. 2021; Soares 2005; Torche and Rauf 2021). Indeed, as more people hunker down to care for sick infants (Dyregrov and Matthiesen 1991; George et al. 2007), the fewer opportunities there are for civic participation, day-to-day interactions among neighbors, and sharing information about job opportunities, training, and other resources with others in the community, independent of health concerns. In the aggregate, the quality of social life in a community and opportunities for others to find jobs and other related resources are diminished. Indeed, research has shown how important neighbors and acquaintances can be for job hunting and employment (Granovetter 1973; Hellerstein et al. 2011, 2014; Lin and Dumin 1986; Marin 2012). On the positive side, our results extend existing knowledge by suggesting that addressing infant health problems can structurally benefit entire communities.

Second, we found that dissimilarities in infant health problems were significantly associated with a decreased likelihood of ties between any two communities during the study. In contrast with a large body of knowledge in medical sociology indicating the role of social and economic adversity and inequality in shaping differences in infant health (Conley and Springer 2001; Light and Marshall 2018; Torche 2011; Wildeman 2012), the current study is the first to show evidence on the reverse pathway, that infant health differences significantly exacerbate labor market inequalities among communities. The findings suggest that preexisting health dissimilarities contribute over time to separating segments of the city further apart, expanding existing knowledge on the significance of population health in reinforcing and deepening existing social inequalities and spatial divisions (Conley and Springer 2001; Torche and Rauf 2021). The findings also add important new evidence to a growing body of work on econetworks and health (Browning et al. 2015; Browning, Calder, Boettner, and Smith 2017) and highlight for the first time the relevance of infant health in shaping a community’s econetwork location in the citywide labor market and the unequal connectivity patterns among communities in a large U.S. city.

Overall, the results of this study highlight two key ways in which addressing health problems can have broad implications not just for individuals but also for entire communities and ultimately, for the entire city. First, programs and policies that address infant health burdens at the community level may slow down, stop, or even reverse the downward spiral of structural isolation of communities. Policy makers and employers may reduce employees’ commuting burdens from health issues by perhaps encouraging and supporting flexible work arrangements, family leave policies, and transportation programs (Gee and Takeuchi 2004; Kahneman et al. 2004; Lourel et al. 2009; McCormick et al. 1991; Stringer 1998).

Second, the current findings suggest that programs focused on addressing infant health may also support the development of a more widely connected infrastructure across the city. As our networks’ geographic visualizations indicate, many ties between communities of different levels of infant health do exist already. While they do not represent the dominant pattern, their presence offers hope that cross-cutting connections are possible. They present windows of opportunity for future research and policies to explore how such connections may be further strengthened and expanded, enabling a freer flow of populations across space, less dependent on preexisting social or spatial proximity. In doing so, they would allow for a more equitable match of labor to existing structural opportunities.

## Supplemental Material

sj-docx-1-hsb-10.1177_00221465231172176 – Supplemental material for The Role of Infant Health Problems in Constraining Interneighborhood Mobility: Implications for Citywide Employment Networks Click here for additional data file. Supplemental material, sj-docx-1-hsb-10.1177_00221465231172176 for The Role of Infant Health Problems in Constraining Interneighborhood Mobility: Implications for Citywide Employment Networks by Megan Evans, Corina Graif and Stephen A. Matthews in Journal of Health and Social Behavior

## Notes

1. Over 21,000 infants died before 1 year of age in 2018 alone (about 5.7 deaths per 1000 live births).

2. Throughout the article, we use the terms “community” and “neighborhood” interchangeably. Because we are studying the city of Chicago, we use these terms to also refer to the city’s well-defined residential Community Areas (Sampson 2012).

3. Only focusing on Chicago’s 77 Community Areas, we excluded commuting ties to areas outside the city.

4. Smaller thresholds led most of the communities to be connected to one another, while higher thresholds led communities to be connected to only a few hub work communities.

5. Chicago had an infant mortality rate in 2015 of 7.9, comparable with Baltimore, another segregated city (8.4), but higher than the U.S. average (5.9), New York City (4.1), and Los Angeles (3.9) and lower than Detroit (14.4). The average travel time to work in the Chicago metro area in 2019 was 32.4 minutes, lower than the New York City metro ( 37.7 minutes) but similar to Los Angeles and Baltimore areas (both at 31.7 minutes) and higher than Detroit area (27.5 minutes) or the average U.S. commuting time (27.6 minutes).

## References

[bibr1-00221465231172176] Abowd John M. Vilhuber Lars . 2011. “National Estimates of Gross Employment and Job Flows from the Quarterly Workforce Indicators with Demographic and Industry Detail.” Journal of Econometrics 161(1):82–99. 21516213 10.1016/j.jeconom.2010.09.008 PMC3079891

[bibr2-00221465231172176] Alavinia Seyed Mohammad Molenaar Duco Burdorf Alex . 2009. “Productivity Loss in the Workforce: Associations with Health, Work Demands, and Individual Characteristics.” American Journal of Industrial Medicine 52(10):49–56. 18942667 10.1002/ajim.20648

[bibr3-00221465231172176] Alderman Harold Behrman Jere R . 2006. “Reducing the Incidence of Low Birth Weight in Low-Income Countries Has Substantial Economic Benefits.” World Bank Research Observer 21(1):25–48.

[bibr4-00221465231172176] Alexander Monica Root Leslie . 2022. “Competing Effects on the Average Age of Infant Death.” Demography 59(2):587–605. 35244673 10.1215/00703370-9779784

[bibr5-00221465231172176] Arcaya Mariana C. Graif Corina Waters Mary C. Subramanian S. V. 2015. “Health Selection into Neighborhoods among Families in the Moving to Opportunity Program.” American Journal of Epidemiology 183(2):130–37. 10.1093/aje/kwv189 PMC4706680 26656481

[bibr6-00221465231172176] Behrman Jere R. Rosenzweig Mark R. 2004. “Returns to Birthweight.” Review of Economics and Statistics 86(2):586–601.

[bibr7-00221465231172176] Blair-Loy Mary Wharton Amy S. 2002. “Employees’ Use of Family-Responsive Policies and the Social Context of Work.” Social Forces 80(3):1–30.

[bibr8-00221465231172176] Blumenshine Philip. Egerter Susan Barclay Colleen J. Cubbin Catherine Braceman Paula A. 2010. “Socioeconomic Disparities in Adverse Birth Outcomes: A Systematic Review.” American Journal of Preventative Medicine 39(3):263–72. 10.1016/j.amepre.2010.05.012 20709259

[bibr9-00221465231172176] Braveman Paula A. Egerter Susan A. Mockenhaupt Robin E . 2011. “Broadening the Focus: The Need to Address the Social Determinants of Health.” American Journal of Preventative Medicine 40(Suppl. 1):S4–18. 10.1016/j.amepre.2010.10.002 21146778

[bibr10-00221465231172176] Browning Christopher R. Calder Catherine A. Boettner Bethany Smith Anne . 2017. “Ecological Networks and Urban Crime: The Structure of Shared Routine Activity Locations and Neighborhood-Level Informal Control Capacity.” Criminology 55(4):754–78. 10.1111/1745-9125.12152 PMC5815399 29459884

[bibr11-00221465231172176] Browning Christopher R. Calder Catherine A. Soller Brian Jackson Aubrey L. Dirlam Jonathan . 2017. “Ecological Networks and Neighborhood Social Organization.” American Journal of Sociology 122(6):1939–88. 10.1086/691261 PMC5786432 29379218

[bibr12-00221465231172176] Browning Christopher R. Soller Brian Jackson Aubrey L. 2015. “Neighborhoods and Adolescent Health-Risk Behavior: An Ecological Network Approach.” Social Science & Medicine 125:163–72. 10.1016/j.socscimed.2014.06.028 PMC4272333 25011958

[bibr13-00221465231172176] Burgard Sarah A. Kalousova Lucie . 2015. “Effects of the Great Recession: Health and Well-Being.” Annual Review of Sociology 41:181–201.

[bibr14-00221465231172176] Burgard Sarah A. Lin Katherine Y. 2013. “Bad Jobs, Bad Health? How Work and Working Conditions Contribute to Health Disparities.” American Behavior Scientists 57(8):1105–27. 10.1177/0002764213487347 PMC3813007 24187340

[bibr15-00221465231172176] Bursik Robert J. Grasmick Harold G. 1999. Neighborhoods and Crime: The Dimensions of Effective Community Control. New York, NY: Lexington Books.

[bibr16-00221465231172176] Bütikofer Aline Løken Katrine V. Salvanes Kjell G. 2019. “Infant Health Care and Long-Term Outcomes.” Review of Economics and Statistics 101(2):341–54.

[bibr17-00221465231172176] Carpiano Richard M . 2006. “Toward a Neighborhood Resource–Based Theory of Social Capital for Health: Can Bourdieu and Sociology Help?” Social Science & Medicine 62(1):165–75. 10.1016/j.socscimed.2005.05.020 15992978

[bibr18-00221465231172176] CDC. 2020. Maternal and Infant Health 2019. Atlanta, GA: U.S. Department of Health and Human Services.

[bibr19-00221465231172176] City of Chicago. 2019. “Chicago Data Portal.” June 6. https://data.cityofchicago.org/.

[bibr20-00221465231172176] Conley Dalton Springer Kristen W. 2001. “Welfare State and Infant Mortality.” American Journal of Sociology 107(3):768–807. 10.1086/338781 12109504

[bibr21-00221465231172176] Conley Dalton Strully Kate Wetteroth Bennett Neil G. 2003. The Starting Gate: Birth Weight and Life Chances. Berkeley: University of California Press.

[bibr22-00221465231172176] Cuevas Katherine D. Silver Debra R. Brooten Dorothy Youngblut JoAnne M. Bobo Charles M. 2005. “Hospital Charges at Birth and Frequency of Rehospitalizations and Acute Care Visits over the First Year of Life: A Comparison by Gestational Age and Birth Weight.” The American Journal of Nursing 105(7):56–65. 10.1097/00000446-200507000-00031 PMC3575194 15995395

[bibr23-00221465231172176] Culhane Jennifer F. Elo Irma T . 2005. “Neighborhood Context and Reproductive Health.” American Journal of Obstetrics and Gynecology 192(5S):S22–29. 10.1016/j.ajog.2005.01.071 15891708

[bibr24-00221465231172176] De Menezes Lilian M. Kelliher Clare . 2017. “Flexible Working, Individual Performance, and Employee Attitudes: Comparing Formal and Informal Arrangements.” Human Resource Management 56(6):1051–70.

[bibr25-00221465231172176] Dunkel Schetter Christine . 2011. “Psychological Science on Pregnancy: Stress Processes, Biopsychosocial Models, and Emerging Research Issues.” Annual Review of Psychology 62:531–58. 10.1146/annurev.psych.031809.130727 21126184

[bibr26-00221465231172176] Dyregrov Atle Matthiesen Stig Berge . 1991. “Parental Grief Following the Death of an Infant—A Follow-Up over One Year.” Scandinavian Journal of Psychology 32(3):193–207. 10.1111/j.1467-9450.1991.tb00869.x 1759138

[bibr27-00221465231172176] Galaskiewicz Joseph Shatin Deborah . 1981. “Leadership and Networking among Neighborhood Human Service Organizations.” Administrative Science Quarterly 26(3):434–48.

[bibr28-00221465231172176] Gee Gilbert C. Takeuchi David T . 2004. “Traffic Stress, Vehicular Burden, and Well-Being: A Multilevel Analysis.” Social Science & Medicine 59(2):405–14. 10.1016/j.socscimed.2003.10.027 15110429

[bibr29-00221465231172176] George Ajesh Vickers Margaret H. Wilkes Lesley Barton Belinda . 2007. “Chronic Grief: Experiences of Working Parents of Children with Chronic Illness.” Contemporary Nurse 23(2):228–42. 10.5555/conu.2006.23.2.228 17343526

[bibr30-00221465231172176] Gobillon Laurent Selod Harris Zenou Yves . 2007. “The Mechanisms of Spatial Mismatch.” Urban Studies 44(12):2401–27.

[bibr31-00221465231172176] Gonzalez Robert M. Gilleskie Donna . 2017. “Infant Mortality Rate as a Measure of a Country’s Health: A Robust Method to Improve Reliability and Comparability.” Demography 54(2):701–20. 10.1007/s13524-017-0553-7 PMC6681443 28233234

[bibr32-00221465231172176] Gorman Bridget K . 1999. “Racial and Ethnic Variation in Low Birthweight in the United States: Individual and Contextual Determinants.” Health & Place 5(3):195–207. 10984575 10.1016/s1353-8292(99)00009-x

[bibr33-00221465231172176] Graham Matthew R. Kutzbach Mark J. McKenzie Brian , 2014. “Design Comparison of LODES and ACS Commuting Data Products.” Center for Economic Studies, U.S. Census Bureau, Working Paper No. 14-38.

[bibr34-00221465231172176] Graif Corina Gladfelter Andrew S. Matthews Stephen A . 2014. “Urban Poverty and Neighborhood Effects on Crime: Incorporating Spatial and Network Perspectives.” Sociology Compass 8(9):1140–55. 10.1111/soc4.12199 PMC4928692 27375773

[bibr35-00221465231172176] Graif Corina Lungeanu Alina Yetter Alyssa M. 2017. “Neighborhood Isolation in Chicago: Violent Crime Effects on Structural Isolation and Homophily in Interneighborhood Commuting Networks.” Social Networks 51:40–59. 29104357 10.1016/j.socnet.2017.01.007 PMC5663310

[bibr36-00221465231172176] Granovetter Mark S ., 1973. “The Strength of Weak Ties.” American Journal of Sociology 78(6):1360–80.

[bibr37-00221465231172176] Grice Mira M. McGovern Patricia Alexander Bruce H. 2008. “Flexible Work Arrangements and Work–Family Conflict after Childbirth.” Occupational Medicine 58(7):468–74. 10.1093/occmed/kqn090 18667390

[bibr38-00221465231172176] Grusky David B. Western Bruce Wimer Christopher , eds. 2011. The Great Recession. New York, NY: Russell Sage Foundation.

[bibr39-00221465231172176] Halpern Diane F . 2005. “How Time-Flexible Work Policies Can Reduce Stress, Improve Health, and Save Money.” Stress and Health 21(3):157–68.

[bibr40-00221465231172176] Hellerstein Judith K. Kutzbach Mark Neumark David . 2014. “Do Labor Market Networks Have an Important Spatial Dimension?” Journal of Urban Economics 79(6):39–58.

[bibr41-00221465231172176] Hellerstein Judith K. McInerney Melissa Neumark David . 2011. “Neighbors and Coworkers: The Importance of Residential Labor Market Networks.” Journal of Labor Economics 29(4):659–95.

[bibr42-00221465231172176] Holt Helle Thaulow Ivan . 1996. “Formal and Informal Flexibility in the Workplace.” Pp. 79–92 in The Work–Family Challenge: Rethinking Employment, edited by Lewis S. Lewis J. London: SAGE.

[bibr43-00221465231172176] Hunter Albert D . 1985. “Private, Parochial and Public Social Orders: The Problem of Crime and Incivility in Urban Communities.” Pp 230–42 in The Challenge of Social Control, edited by Suttles G. Zald M. Norwood, NJ: Ablex.

[bibr44-00221465231172176] Hunter David R. 2007. “Curved Exponential Family Models for Social Networks.” Social Networks 29(2):216–30. 10.1016/j.socnet.2006.08.005 PMC2031865 18311321

[bibr45-00221465231172176] Hunter David R. Handcock Mark S. Butts Carter T. Goodreau Steven M. Morris Martina . 2008. “ergm: A Package to Fit, Simulate and Diagnose Exponential-Family Models for Networks.” Journal of Statistical Software 24(3):nihpa54860. doi:10.18637/jss.v024.i03. PMC2743438 19756229

[bibr46-00221465231172176] Ibarra Herminia . 1995. “Race, Opportunity, and Diversity of Social Circles in Managerial Networks.” Academy of Management Review 38(3):673–703.

[bibr47-00221465231172176] Kahneman Daniel Krueger Alan B. Schkade David A. Schwarz Norbert Stone Arthur A. 2004. “A Survey Method for Characterizing Daily Life Experience: The Day Reconstruction Method.” Science 306(5702):1776–80. 10.1126/science.1103572 15576620

[bibr48-00221465231172176] Kain John F . 1968. “Housing Segregation, Negro Employment, and Metropolitan Decentralization.” Quarterly Journal of Economics 82(2):175–97.

[bibr49-00221465231172176] Kain John F . 2004. “A Pioneer’s Perspective on the Spatial Mismatch Literature.” Urban Studies 41(1):7–32.

[bibr50-00221465231172176] Kalemli-Ozcan Sebnem . 2002. “Does the Mortality Decline Promote Economic Growth?” Journal of Economic Growth 7(4):411–39.

[bibr51-00221465231172176] Kalleberg Arne L. von Wachter Till M. 2017. “The U.S. Labor Market during and after the Great Recession: Continuities and Transformations.” RSF: The Russell Sage Foundation Journal of the Social Sciences 3(3):1–19. 29780882 10.7758/rsf.2017.3.3.01 PMC5959048

[bibr52-00221465231172176] Kawachi Ichiro Kennedy Bruce P. Lochner Kimberly Prothrow-Stith Deborah . 1997. “Social Capital, Income Inequality, and Mortality.” American Journal of Public Health 87(9):1491–98. 10.2105/ajph.87.9.1491 PMC1380975 9314802

[bibr53-00221465231172176] Kawachi Ichiro Subramanian S. V. Kim Daniel , eds. 2008. Social Capital and Health. New York, NY: Springer.

[bibr54-00221465231172176] Kelling Claire Graif Corina Korkmaz Gizem Haran Murali . 2021. “Modeling the Social and Spatial Proximity of Crime: Domestic and Sexual Violence across Neighborhoods.” Journal of Quantitative Criminology 37(2):481–516. 34149156 10.1007/s10940-020-09454-w PMC8210633

[bibr55-00221465231172176] Kessler Ronald C. Greenberg Paul E. Mickelson Kristin D. Meneades Laurie M. Wang Philip S. 2001. “The Effects of Chronic Medical Conditions on Work Loss and Work Cutback.” Journal of Occupational and Environmental Medicine 43(3):218–25. 10.1097/00043764-200103000-00009 11285869

[bibr56-00221465231172176] Kim Kyusik Horner Mark . 2021. “Examining the Impacts of the Great Recession on the Commuting Dynamics and Jobs–Housing Balance of Public and Private Sector Workers.” Journal of Transport Geography 90:102933. doi:10.1016/j.jtrangeo.2020.102933.

[bibr57-00221465231172176] Koval John P. Bennett Larry Bennett Michael J. Demissie Fassil Garner Roberta Kim Kiljoong , eds. 2006. The New Chicago: A Social and Cultural Analysis. Philadelphia, PA: Temple University Press.

[bibr58-00221465231172176] Krivitsky Pavel N. Handcock Mark . 2019. “tergm: Fit, Simulate, and Diagnose Models for Network Evolution Based on Exponential-Family Random Graph Models.” The Statnet Project. R Package, Version 3. https://statnet.org.

[bibr59-00221465231172176] Leifeld Philip Cranmer Skyler J. Desmarais Bruce A. 2017. “xergm: Extensions of Exponential Random Graph Models.” R Package Version 1.8.2. https://rdrr.io/cran/xergm/

[bibr60-00221465231172176] Levy Brian L. Phillips Nolan E. Sampson Robert J. 2020. “Triple Disadvantage: Neighborhood Networks of Everyday Urban Mobility and Violence in U. S. Cities.” American Sociological Review 85(6):925–56.

[bibr61-00221465231172176] Light Michael T. Marshall Joey . 2018. “On the Weak Mortality Returns of the Prison Boom: Comparing Infant Mortality and Homicide in the Incarceration Ledger.” Journal of Health and Social Behavior 59(1):3–19. 29283677 10.1177/0022146517748412

[bibr62-00221465231172176] Lin Nan Dumin Mary . 1986. “Access to Occupations through Social Ties.” Social Networks 8(4):365–85.

[bibr63-00221465231172176] Logan John R. Molotch Harvey Luskin . 1987. Urban Fortunes: The Political Economy of Place. Berkeley: University of California Press.

[bibr64-00221465231172176] Lourel Marcel Ford Michael T. Edey Claire Guéguen Nicolas Hartmann Anne . 2009. “Negative and Positive Spillover between Work and Home: Relationship to Perceived Stress and Job Satisfaction.” Journal of Managerial Psychology 24(5):438–49.

[bibr65-00221465231172176] Lyons Christopher J. Vélez María B. Santoro Wayne A. 2013. “Neighborhood Immigration, Violence, and City-Level Immigrant Political Opportunities.” American Sociological Review 78(4):604–32.

[bibr66-00221465231172176] Marin Alexandra . 2012. “Don’t Mention It: Why People Don’t Share Job Information, When They Do, and Why It Matters.” Social Networks 34(2):181–92.

[bibr67-00221465231172176] Marsden Peter V . 1988. “Homogeneity in Confiding Relations.” Social Networks 10(1):57–76.

[bibr68-00221465231172176] McCormick Marie C. Bembaum Judy C. Eisenberg John M. Kustra Sharon Lee Finnegan Emily . 1991. “Costs Incurred by Parents of Very Low Birth Weight Infants after the Initial Neonatal Hospitalization.” Pediatrics 88(3):533–41. 1652734

[bibr69-00221465231172176] McMillan Cassie . 2019. “Tied Together: Adolescent Friendship Networks, Immigrant Status, and Health Outcomes.” Demography 56(3):1075–103. 10.1007/s13524-019-00770-w PMC7334874 30887310

[bibr70-00221465231172176] McPherson Miler Smith-Lovin Lynn Cook James M. 2001. “Birds of a Feather: Homophily in Social Networks.” Annual Review of Sociology 27:415–44.

[bibr71-00221465231172176] Morenoff Jeffrey D . 2003. “Neighborhood Mechanisms and the Spatial Dynamics of Birth Weight.” American Journal of Sociology 108(5):976–1017. 10.1086/374405 14560732

[bibr72-00221465231172176] Morris Janowitz . 1979. “Social Inequalities Undiminished.” Lancet 313(8107):87–90. 10.1016/s0140-6736(79)90073-4 84138

[bibr73-00221465231172176] Moster Dag Lie Rolv Terje Markestad Trond . 2008. “Long-Term Medical and Social Consequences of Preterm Birth.” New England Journal of Medicine 359(3):262–73. 10.1056/NEJMoa0706475 18635431

[bibr74-00221465231172176] Neal Zachary P . 2013. The Connected City: How Networks Are Shaping the Modern Metropolis. New York, NY: Routledge.

[bibr75-00221465231172176] Newmyer Lauren Evans Megan Graif Corina . 2022. “Socially Connected Neighborhoods and the Spread of Sexually Transmitted Infections.” Demography 59(4):1299–323. 10.1215/00703370-10054898 PMC9707946 35838157

[bibr76-00221465231172176] OECD. 2022. “Gross Domestic Product (GDP) Indicator.” https://www.americashealthrankings.org/learn/reports/2018-annual-report/findings-inter national-comparison.

[bibr77-00221465231172176] Papachristos Andrew V. Bastomski Sara . 2018. “Connected in Crime: The Enduring Effect of Neighborhood Networks on the Spatial Patterning of Violence.” American Journal of Sociology 124(2):517–68.

[bibr78-00221465231172176] Petrou Stavros Mehta Ziyah Hockley Christine Cook-Mozaffari Paula Henderson Jane Goldacre Michael . 2003. “The Impact of Preterm Birth on Hospital Inpatient Admissions and Costs during the First 5 Years of Life.” Pediatrics 112(6 Pt 1):1290–97. 10.1542/peds.112.6.1290 14654599

[bibr79-00221465231172176] Putnam Robert . 2000. Bowling Alone: The Collapse and Revival of American Community. New York, NY: Simon and Schuster.

[bibr80-00221465231172176] Reidpath Daniel D. Allotey Pascale . 2003. “Infant Mortality Rate as an Indicator of Population Health.” Journal of Epidemiology & Community Health 57(5):344–46. 10.1136/jech.57.5.344 PMC1732453 12700217

[bibr81-00221465231172176] Roberts Jennifer Hodgson Robert Dolan Paul . 2011. “It’s Driving Her Mad: Gender Differences in the Effects of Commuting on Psychological Well-Being.” Journal of Health Economics 30(5):1064–76. 10.1016/j.jhealeco.2011.07.006 21855154

[bibr82-00221465231172176] Rocco Lorenzo Fumagalli Elena Mirelman Andrew J. Suhrcke Marc . 2021. “Mortality, Morbidity, and Economic Growth.” PLoS One 16(5):e0251424. doi:10.1371/journal.pone.0251424. PMC8158917 34043654

[bibr83-00221465231172176] Sampson Robert J . 2012. Great American City: Chicago and the Enduring Neighborhood Effect. Chicago, IL: The University of Chicago Press.

[bibr84-00221465231172176] Sampson Robert J. Graif Corina . 2009. “Neighborhood Social Capital as Differential Social Organization: Resident and Leadership Dimensions.” American Behavioral Scientist 52(11):1579–605.

[bibr85-00221465231172176] Sampson Robert J. Sharkey Patrick . 2008. “Neighborhood Selection and the Social Reproduction of Concentrated Racial Inequality.” Demography 45(1):1–29. 18390289 10.1353/dem.2008.0012 PMC2831380

[bibr86-00221465231172176] Schaefer David R . 2012. “Youth Co-offending Networks: An Investigation of Social and Spatial Effects.” Social Networks 34(1):141–49.

[bibr87-00221465231172176] Shaw Clifford R. McKay Henry D . 1942. Juvenile Delinquency and Urban Areas: A Study of Rates of Delinquency in Relation to Differential Characteristics of Local Communities in American Cities. Chicago, IL: The University of Chicago Press.

[bibr88-00221465231172176] Siddiqi Arjumand Jones Marcella K. Bruce Donald J. Erwin Paul C. 2016. “Do Racial Inequities in Infant Mortality Correspond to Variations in Societal Conditions? A Study of State-Level Income Inequality in the U.S., 1992–2007.” Social Science & Medicine 164:49–58. 27471130 10.1016/j.socscimed.2016.07.013

[bibr89-00221465231172176] Sloan Melissa M. Evenson Newhouse Ranae J. Thompson Ashley B. 2013. “Counting on Coworkers: Race, Social Support, and Emotional Experiences on the Job.” Social Psychology Quarterly 76(4):343–72.

[bibr90-00221465231172176] Soares Rodrigo R . 2005. “Mortality Reductions, Educational Attainment, and Fertility Choice.” American Economic Review 95(3):580–601. 29125724 10.1257/0002828054201486

[bibr91-00221465231172176] Stringer M . 1998. “Personal Costs Associated with High-Risk Prenatal Care Attendance.” Journal of Health Care 9(3):222–35. 10.1353/hpu.2010.0248 10073205

[bibr92-00221465231172176] Tommiska Viena Tuominen Risto Fellman Vineta . 2003. “Economic Costs of Care in Extremely Low Birthweight Infants during the First 2 Years of Life.” Pediatric Critical Care Medicine 4(2):157–63. 10.1097/01.PCC.0000059731.74435.02 12749645

[bibr93-00221465231172176] Torche Florencia . 2011. “The Effect of Maternal Stress on Birth Outcomes: Exploiting a Natural Experiment.” Demography 48(4):1473–91. 10.1007/s13524-011-0054-z 21870187

[bibr94-00221465231172176] Torche Florencia Rauf Tamkinat . 2021. “The Political Context and Infant Health in the United States.” American Sociological Review 86(3):377–405.

[bibr95-00221465231172176] U.S. Bureau of Labor Statistics. 2010. “Metropolitan Area Employment and Unemployment (Monthly) News Release.” https://www.bls.gov/news.release/archives/metro_02022010.htm.

[bibr96-00221465231172176] U.S. Census Bureau. 2000. “Decennial Census Estimates, 2000.” https://www.socialexplorer.com/solutions-education-for-librarians

[bibr97-00221465231172176] U.S. Census Bureau. 2012. “American Community Survey 5-Year Estimates, 2008–2012.” https://www.socialexplorer.com/solutions-education-for-librari ans.

[bibr98-00221465231172176] Wildeman Christopher . 2012. “Imprisonment and Infant Mortality.” Social Problems 59(2):228–57.

[bibr99-00221465231172176] Williams David R . 2002. “Racial-Ethnic Variations in Women’s Health: The Social Embeddedness of Health.” American Journal of Public Health 92(4):588–97. 10.2105/ajph.92.4.588 PMC1447123 11919058

