# Supplementary material for: The Role of Infant Health Problems in Constraining Interneighborhood Mobility: Implications for Citywide Employment Networks
Source: J Health Soc Behav. 2023 Jun 4;64(4):555–77. doi: 10.1177/00221465231172176 (PMC10683334; doi:10.1177/00221465231172176)
Supplement: sj-docx-1-hsb-10.1177_00221465231172176 – Supplemental material for The Role of Infant Health Problems in Constraining Interneighborhood Mobility: Implications for Citywide Employment Networks [file sj-docx-1-hsb-10.1177_00221465231172176.docx]

**Journal** of **Health**

and **Social Behavior**

OFFICIAL JOURNAL OF THE AMERICAN SOCIOLOGICAL ASSOCIATION

**ONLINE SUPPLEMENT**

**to article in**

Journal of Health and Social Behavior

**The Role of Infant Health Problems in Constraining Inter-neighborhood Mobility: Implications for Citywide Employment Networks**

**Megan Evans**

*Pennsylvania State University*

**Corina Graif**

*Pennsylvania State University*

**Stephen A. Matthews**

*Pennsylvania State University*

**List of Appendices**

Appendix A: Explanation of TERGM

Appendix B: Assessing Goodness of Fit

Figure B.1: Goodness-of-Fit Statistics for TERGM Table 2 Model 2

Appendix C: Assessing Potential Confounders

Table C.1: Bootstrapped Temporal Exponential Random Graph Models Assessing Work-Related Confounders, 2002 to 2015

Table C.2: Bootstrapped Temporal Exponential Random Graph Models Assessing Health-Related Confounders, 2002 to 2015

Table C.3: Bootstrapped Temporal Exponential Random Graph Models Assessing SES-Related Confounders, 2002 to 2015

Table C.4 Bootstrapped Temporal Exponential Random Graph Models Assessing Most Confounders Together, 2002 to 2015

Appendix D: Assessing Linear Interpolation of Infant Health

Table D.1: Bootstrapped Temporal Exponential Random Graph Models Assessing Non-Interpolated Health Problems, 2002, 2007, and 2012

Appendix E: Assessing Commuting Population Thresholds

Table E.1: Averaged Descriptive Statistics Assessing Commuting Threshold, 2002 to 2015

Table E.2: Bootstrapped Temporal Exponential Random Graph Models Assessing Different Commuting Tie Cutoffs of CA Population, 2002 to 2015

Table E.3: Bootstrapped Temporal Exponential Random Graph Models Assessing Statistically Significant Local Commuting Tie Cutoff using the Disparity Filter, 2002 to 2015

Appendix F: Assessing Reverse Causality

Table F.1 Bootstrapped Temporal Exponential Random Graph Models Assessing Commuting and Dissimilarity in Infant Health Problems, 2002 to 2015

Appendix G: Black and White Figures

Figure G.1. Black and White Version of Figure 1

Figure G.2. Black and White Version of Figure 2

Figure G.3. Black and White Version of Figure 3

Appendix H: References

## *Appendix A: Explanation of ERGM*

We include several measures in our models to account for the structural properties of the commuting network. The number of edges represents the network density, or the degree of commuting which occurs among the communities in the network. Reciprocity represents the number of mutual ties in the network and is considered a common structure of complex directed networks (Wasserman and Faust 2010). The geometrically weighted indegree distribution accounts for the popularity spread of the network, i.e., if certain communities receive more incoming commuting ties than others. Accounting for the geometrically weighted indegree distribution helps us account for network centralization, a common feature of networks where several nodes receive a majority of incoming ties, i.e., are more popular (Hunter 2007; Levy 2016; Snijders et al. 2006). Finally, the geometrically weighted number of edgewise shared partners represents the number of closed triangles and the geometrically weighted number of dyadic shared partners represents the number of open triangles. Closed triangles represent transitivity and open triangles represent the preconditions for transitivity, both common features of complex networks (Hunter 2007; Snijders et al. 2006).

The ERGM, and its longitudinal extension, the TERGM, predicts the probability of pairwise patterns in the given network (Graif et al. 2017; Hunter 2007; Leifeld et al. 2017; McMillan 2019). ERGMs use Markov chain Monte Carlo (MCMC) to compare the current network to a sample of random networks taken from a probability distribution. Fitting this random algorithm allows the ERGM to estimate the true likelihood function and assess if any of our network structures are more or less likely to occur than chance (Hunter 2007). By providing us the log likelihood that certain network statistics are more or less likely to occur, ERGM terms can be interpreted as odds ratios after being exponentiated (Leifeld et al. 2017; Robins et al. 2007). Positive and significant coefficients indicate that the respective network structures are more likely to occur than by random chance, while negative and significant coefficients indicate that the respective network structures are less likely to occur than by random chance.

While an ERGM would allow us to examine each of our yearly commuting networks cross-sectionally, a TERGM allows us to examine how our network evolves over time. With a TERGM we can analyze what network structures predict the existence of ties across our fourteen-year period, accounting for inter-temporal dependence. We estimate our TERGM using maximum pseudolikelihood with bootstrapped (bootstrapping sample size=1000) confidence intervals using the *xergm* package in R (Leifeld et al. 2017). If the linear time trend is positive and significant, it would mean that the network gets denser over time. The memory term represents positive autoregression, or whether there is dyadic stability in the commuting ties across time. Hence, a statistically significant positive memory term would indicate that commuting ties present in the year prior are more likely to be present again in the following year than expected by chance.

Building on Leifeld et al. (2017), the TERGM model is represented by the following equation,

$$P\left( N^{K+1}, \ldots, N^{T} \right|N^{1}, \ldots, N^{K}, \theta)= \prod_{t=K+1}^{T} \frac{exp(\theta^{⊺}h\left( N^{t}, N^{t-1}, \ldots, N^{t-K} \right))}{c(\theta,N^{t-K}, \ldots,N^{t-1})}$$

where we are predicting the probability of observing the commuting networks, *N*, between times K+1 and T. In this equation, *N* is the adjacency matrix of our commuting network in which *N_ij_* = 1 if community *i* sends a commuting tie to community *j* and 0 otherwise. We predict the probability of our observed commuting networks by taking the product of the probabilities of the individual networks conditional on the others. The vector of model coefficients is represented by $\theta$, *h*(*N*) represents the vector of statistics accounting for endogenous and exogenous network dependencies, and $c\left( \theta{,N}^{t-K}, \ldots,N^{t-1} \right)$ represents the set of all possible permutations of the network given the same number of nodes.

## *Appendix B: Assessing Goodness of Fit*

To assess the goodness-of-fit for our TERGMs we simulate 1,000 networks generated at random from the specified coefficients in our final model, Model 2 in Table 2. We graph these simulations to examine how our simulated networks align with the actual commuting network. In Figure B.1, the black line shows the actual frequencies of the respective network statistics in our real commuting network overlaid on the boxplot of the values from the simulated networks. We find that our simulated networks match closely to the real network regarding edgewise shared partners, geodesic distance, and degree. This suggests good model fit.

**Figure B.1.** Goodness of Fit Statistics for TERGM Table 2 Model 2

## *Appendix C: Assessing Potential Confounders*

Tables C.1, C.2, C.3, and C.4 present several models assessing whether omitted variable bias explains the relationship between infant health problems and commuting ties. Table C.1 examines omitted variables related to employment, Table C.2 examines omitted variables related to health, and Table C.3 examine omitted variables related to socioeconomic status. Table C.4 includes a comprehensive set of confounders as controls in a single model. In Table C.1, first we account for the density of local workers. We measure the density of local workers using data from the LEHD. We account for the number of persons who work locally using the standardized proportion of the number of jobs located in the community occupied by community residents. The second measure we include is the commercial to residential zoning ratio. We next account for the ratio of workers to residents in a community. Finally, we capture the working age composition of the community by measuring the percentage of the community which is of working age, between 25 and 64; in addition to accounting for the female composition of the working age population.

In Table C.2, we first examine if birth indicators may account for this relationship by controlling for the age-adjusted total fertility rate of each Community Area in addition to the teen birth rate. Both of these variables are standardized. We next examine two alternate community health indicators, an index of adult mortality and community-level years of potential life lost (YPLL). The adult mortality index is a standardized continuous index where higher numbers indicate higher adult mortality rates. The measure is created using ten age-adjusted cause-specific death rates from the City of Chicago’s Data Portal: cancer deaths, heart disease deaths, nephritis nephrotic syndrome and nephrosis deaths, chronic lower respiratory disease deaths, chronic liver disease and cirrhosis deaths, coronary heart disease deaths, Alzheimer’s disease deaths, diabetes-related deaths, diet-related deaths, stroke deaths. This measure is created for each of the six 5-year periods available in the data, which is provided by Epidemiology and Public Health Informatics in the Chicago Department of Public Health (i.e., 2000 to 2004, 2005 to 2009, 2010 to 2014, 2011 to 2015, 2012 to 2016, and 2013 to 2017). The internal reliability of the scale is high (in the range .89-.92 for all periods). The Chicago Department of Public Health uses death certificate data from the Illinois Department of Public Health to calculate YPLL at the community area for each of the five-year time periods indicated above. YPLL represents the total number of persons who died before age 75 divided by the total population under 75 during a specified time period. This measure is then expressed as years of productive life lost per 100,000 population. Both of these alternative measures of community health are standardized with a mean of 0 and standard deviation of 1. Finally, we assess whether the violent crime rate in the community influences our model by taking the total number of violent incidents in a community and dividing it by the number of community residents according to the 2000 decennial census. The violent incidents we include in our rate are homicide, assault, battery, sexual assault, domestic violence, and robbery. We also standardize this measure.

In Table C.3, we assess three measures of socioeconomic status. These measures are created using data from the 2000 Decennial Census and the 2008 to 2012 ACS. The 2008 to 2012 measures are assigned to the year 2010, and we use linear interpolation and extrapolation to obtain values for the missing years. First, we create a measure of unemployment using the percentage of the civilian population in the labor force aged 16 and older that is unemployed. Second, we include the median household income divided by $10,000. Finally, we include a measure for the percentage of the population aged 25 and older with at least a high school education.

In Table C.4 we include a comprehensive set of confounders from Tables C.1, C.2, and C.3 as controls in a single model. Our previous approach was using parsimonious sets of confounders to avoid multicollinearity, as many variables tend to be correlated with each other. In Table C.4, however, to address concerns about spuriousness we run a new model which incorporates most of the confounders in a single model. Specifically, the new models include commercial zoning, female working age population, age-adjusted birth rate, violent crime rate, unemployment, median household income, and population with less than a high school education in the model in addition to the original covariates.

Dissimilarity in infant health problems is a consistent predictor of the commuting network across all model specifications. The models also indicate that dissimilarity in the density of local workers, commercial to residential zoning ratio, worker to resident ratio, female composition of the working age composition, birth rate, teenage birth rate, and high school education influences the likelihood of a commuting tie existing between two communities. These findings suggest that homophily among a range of community-level worker, health, and SES characteristics influence which communities share commuting ties. Interestingly, the models which includes the female composition of the working age population and violent crime rate find a negative coefficient of senders’ infant health, suggesting that infant health problems constrain a community’s ability to send commuters to extra-local jobs. In contrast, the model which includes unemployment finds a positive coefficient of senders’ infant health, suggesting that infant health problems may provide a strong motivating force in pushing commuters to extra-local jobs, despite the health constraints and the hardship of longer commutes. These findings are in contrast with the nonsignificant corresponding coefficient in the main analyses. Even so, all models that consider the potential for omitted variable bias continue to find a significant dissimilarity effect of infant health problems.

## *Appendix D: Assessing Linear Interpolation of Infant Health*

Table D.1 presents models replicating Model 2 in Table 2 assessing non-interpolated and non-overlapping 5-year aggregations of infant health problems. We assign 2002 commuting data to 2000 to 2004 infant health data, 2007 commuting data to 2005 to 2009 infant health data, and 2012 commuting data to 2010 to 2014 infant health data. The model does not yield a sender effect for infant health problems. However, it does yield a dissimilarity effect for infant health. Both patterns are consistent with the main results.

## *Appendix E: Assessing Commuting Population Thresholds*

With valued ties, it is important to consider the value at which a scholar wants to define a relationship as existing (=1) verse a relationship not existing (=0), which is necessary given many complex network models such as TERGMs are not yet able to deal with valued networks, but rather must rely on dichotomized networks. There are two approaches one can use to define a relationship as existing. First, one can choose a global threshold cutoff. Using a global cutoff requires that scholars choose a substantively and/or mathematically meaningful cutoff. In this case, the edges preserved (=1) are those whose weight exceeds a specified threshold. It is considered a ‘global’ model because the same criteria is applied to all edges in the network. It is also often classified as a ‘structural’ model because the decision is based on using structural information from the network, i.e., the edges’ weight (Neal 2022). Second, one can choose a local threshold cutoff. The most widely used local model is the disparity filter (Neal 2022; Serrano, Boguná, and Vespignani 2009). The disparity filter is classified as a ‘local’ model because the model decides to preserve a tie (=1) based on the edge’s importance for the individual node as it compares the edge to the other ties a node has. The model is also classified as a ‘statistical’ model because the model decides to preserve ties (=1) using a statistical null model. More specifically, the disparity filter compares an edge’s observed weight to its expected weight under a null model which expects a node’s total weight to be uniformly distributed across its edges (Serrano, Boguná, and Vespignani 2009). The disparity filter preserves the edges (=1) based on if the observed edge weight is statistically significantly stronger than it is expected to be under the null model. We believe both approaches hold value.

Our main results are presented using the global threshold of 0.5% population cutoff. We approach our justification of this threshold cutoff on two levels. First, we provide more descriptive statistics for our two alternative global threshold cutoffs, in addition to a robust discussion of strong and weak ties to contextualize the context of our decision. Second, we investigate if our results are robust using a local threshold cutoff with the disparity filter specified to a *α* significance level of p<0.05. Overall, our descriptive statistics of varying networks with a global threshold indicate that a 0.5% cutoff is most appropriate. Additionally, when using a local threshold cutoff, we find our findings are robust, with dissimilarity in infant health remaining an important predictor of the commuting network. The consistency of our results with the disparity filter procedure gives us further confidence in our results and definition of a global measure of 0.5% of the population.

***Global Threshold***

When investigating relationships (ties) between any two entities (nodes), network scholars have long been curious about the strength of said relationships. Research has highlighted the importance and varying effects of weak and strong ties on the diffusion of norms, ideas, and information (Aral and Van Alstyne 2011; Granovetter 1973; Larson 2017). Weak ties facilitate simple contagions (e.g., information, resources, behaviors, or disease pathogens) (Centola 2021). Stronger ties, in turn, especially overlapping networks of such ties, are useful for more complex social contagions (e.g., social movements, risky innovations). The extent to which health impacts weaker and stronger commuting ties similarly is an open empirical question. Still, it seems reasonable to expect that weaker ties (ties based on few commuters connecting two neighborhoods) would be more sensitive to disruptions (Centola 2021) such as failing health conditions of families and communities. In contrast, stronger ties (ties based on more commuters connecting two neighborhoods) include more redundancies and thus may be more resistant to such disruptions. Nonetheless, it may be harder for stronger ties to form across larger rather than smaller health divides, while this may be less of an issue for weaker ties.

In Table E.2 we investigate the role of infant health in predicting commuting ties of both a weaker and stronger commuting network than the network used in our main results. We generate a weaker commuting network by defining a commuting tie between two communities if at least 0.25% of the home community’s residents commute to the work community. We create a stronger commuting network by defining a commuting tie between two communities if at least 2% of the home community’s residents commute to the work community.

Table E.1 presents the descriptive statistics. In the weaker commuting network (0.25% population cutoff), the average community sent and received about 15 commuting ties. The minimum number of outgoing commuting ties a community sends into the employment network is 8 while the maximum number is 24. The weaker commuting network has communities connected to twice that of the main commuting network. Among the stronger commuting network (2% population cutoff), the average community sent and received only 2 commuting ties. The contrast in the outdegree statistics is stark. There is now one community that is isolated in the stronger commuting network and sends no outgoing commuting ties, while the maximum number of communities a community sends commuting ties to is 4. In the stronger commuting network, a majority of the communities are only connected to the larger work hubs such as O’Hare. These resulting networks are consistent with a traditional understanding of weak and strong ties (Brashears et al. 2018; Centola and Macy 2007; Granovetter 1973; Murray et al. 1981). The smaller commuting tie threshold leads to many of the communities being connected to each other in the network, while the stronger commuting tie threshold leads most of the communities to be connected to only a select group of communities.

In Table E.2, the results indicate that in the weaker tie commuting network, where a much larger share of communities are connected to one another by a smaller percentage of their residents’ commuting behaviors, poor infant health lowers the likelihood of sending a commuting tie, consistent with our original *neighborhood* *health isolation* hypothesis. These results indicate that communities with higher level of infant health problems are sending fewer commuting ties. However, the dissimilarity effect of infant health problems is no longer significant, which does not support our *health inequality* hypothesis for weak commuting ties.

In the stronger commuting tie network, the role of infant health problems in predicting commuting is consistent with the main results in Table 2. There is a significant dissimilarity effect for infant health problems.

***Local Threshold***

We investigate if our main results using a global threshold of 0.5% are robust using a local threshold cutoff with the disparity filter specified to a *α* significance level of p<0.05. The descriptive statistics offered in Table E.1 indicate that this local threshold creates a commuting network which looks much closer to our main network than either of the stronger or weaker networks investigated above. On average, a community sends and receives 6 commuting ties, 1 less than in our main network.

Table E.3 we replicate our main results from Table 2 and 3 using the commuting network created from the local threshold model. The results are consistent with our main findings. There remains a robust relationship between dissimilarity in infant health and commuting. The only variation is that regarding our infant health isolation hypothesis. Interestingly, we find a significant isolation relationship between infant health and commuting during the pre-recession years but not during or after the recession. These results are not consistent with our main findings which gives us less confidence about our isolation hypothesis. However, there is a robust dissimilarity coefficient across all three models. These findings indicate strong support for our health inequality hypothesis.

## *Appendix F: Assessing Reverse Causality*

For each year of data, we create a valued symmetrical dissimilarity matrix of infant health problems indicating the difference between the level of infant health problems two communities have with each other. We then dichotomize this matrix where communities with above the median value in dissimilarity are considered dissimilar in infant health (=1) and communities with below the median value in dissimilarity are considered similar, or more homophilous, in infant health (=0). We run analyses on a binarized matrix due to the current methodological limitations in the network literature with running longitudinal network analyses on valued networks.

The infant health dissimilarity network is equivalent to one-mode representation of a bipartite, or “affiliation” network. With such networks, a tie does not represent a standard connection between two nodes such as a friendship tie or commuting tie. Rather, two nodes are considered socially proximate to one another (vs socially distant), if they share (or not) a group membership in common. In our case, social proximity and social distance are defined based on having a similar (vs dissimilar) level of infant health problems. We define social similarity based on being more similar in community level infant health problems while we define social dissimilarity based on being more dissimilar in community level infant health problems. With caution against overinterpreting the substantive meaning of this network, it is a valuable way to examine the extent to which the dyadic commuting relationship between communities predicts dissimilarity in infant health problems.

Table F.1 presents the TERGM results predicting dissimilarity in infant health problems over the fourteen-year period. The models present the average effects of our covariates on the log odds of a tie existing, conditional on the rest of the network (Leifeld et al. 2017). In these models the only network structure we control for is edges. The first model only includes terms for edges, relational effects, time effects, and commuting. In Model 2 we include our sociodemographic measures.

Model 1 finds a significant negative effect of commuting, indicating that communities which share commuting ties are more likely to share similar levels of infant health problems. However, when we include controls for all of the community characteristics in Model 2, we find no significant relationship between commuting and dissimilarity in infant health problems. These findings suggest that reverse causality is less of a concern when investigating the relationship between infant health problems and community.

## *Appendix G: Black and White Figures*

Figure G.1. Black and White Version of Figure 1


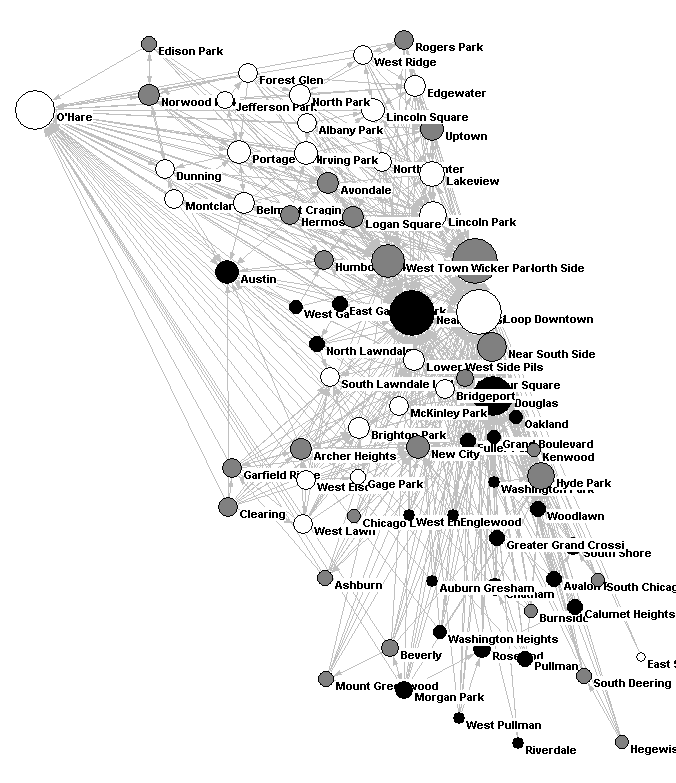

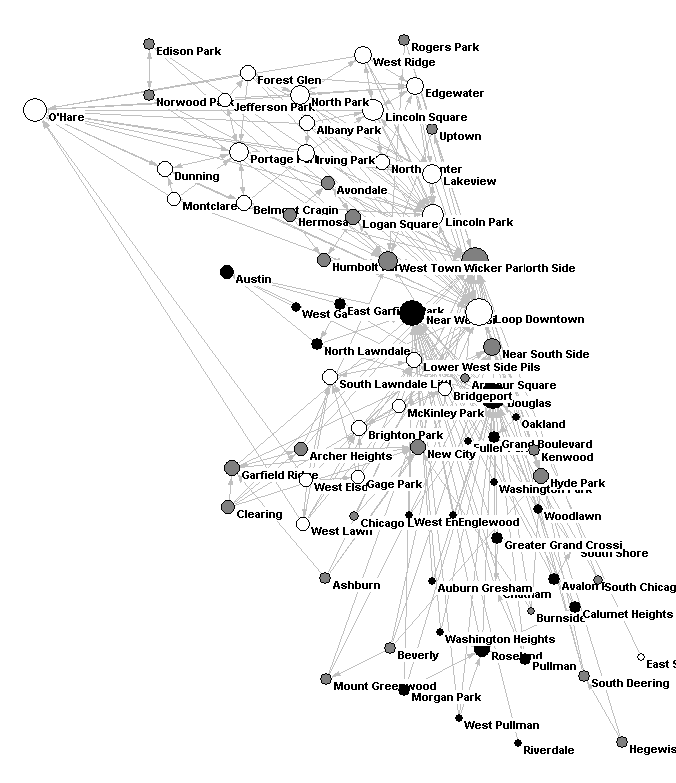

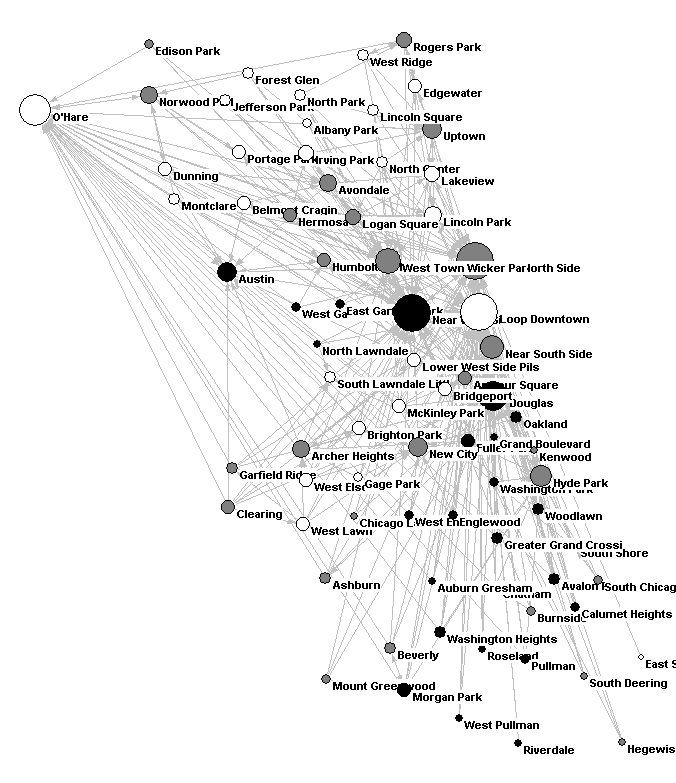


Bottom tercile

**Infant Health Problems**

Middle tercile

Top tercile

A B C

Figure G.2. Black and White Version of Figure 2

*
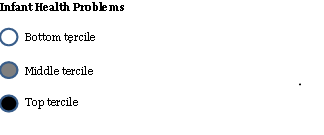
*
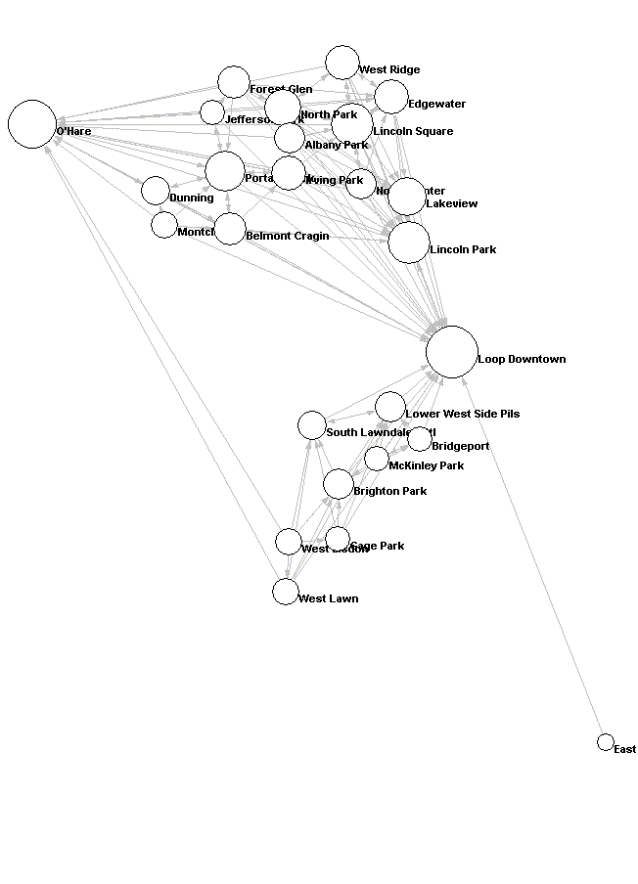

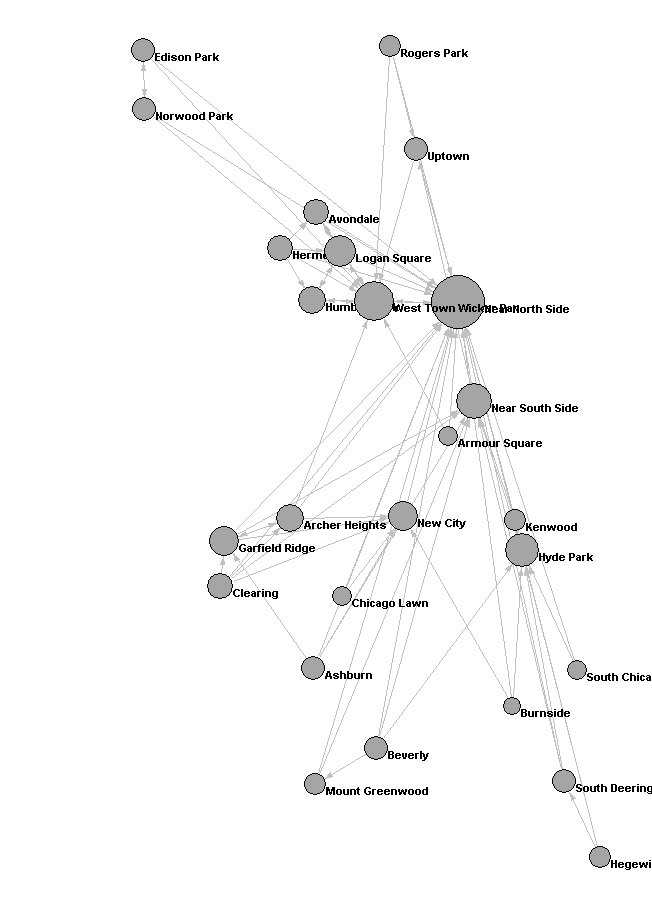

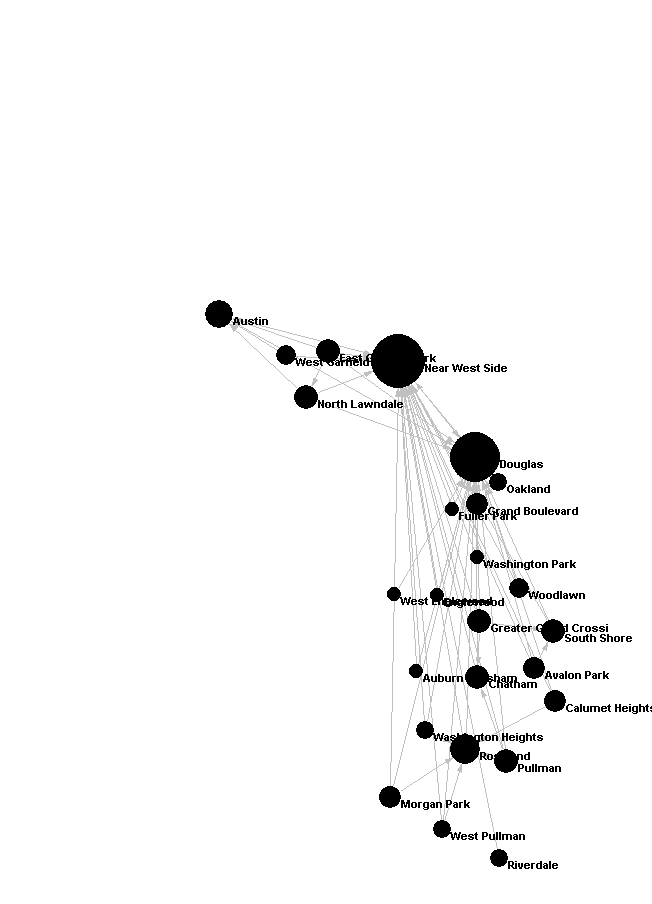


A B C

Figure G.3. Black and White Version of Figure 3


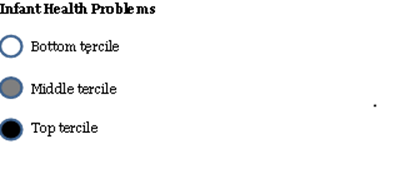

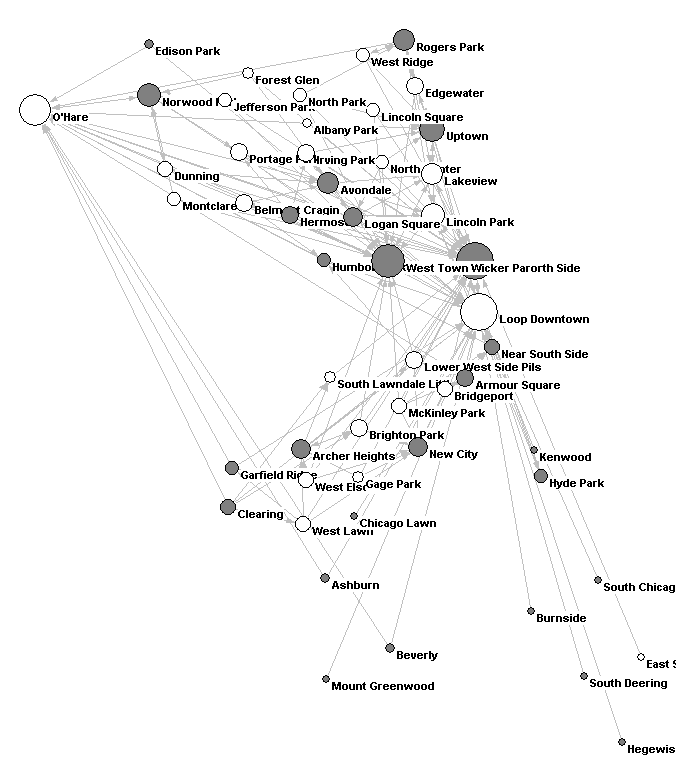

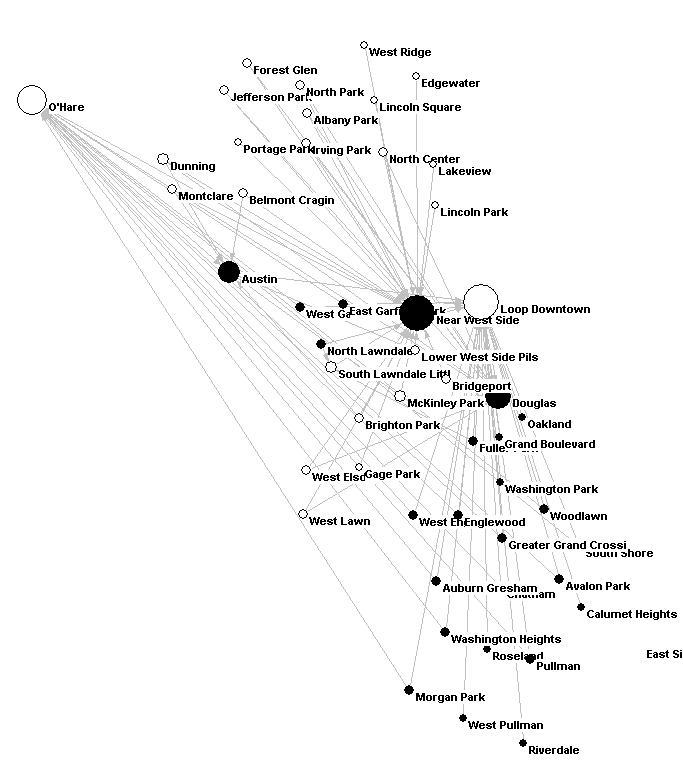

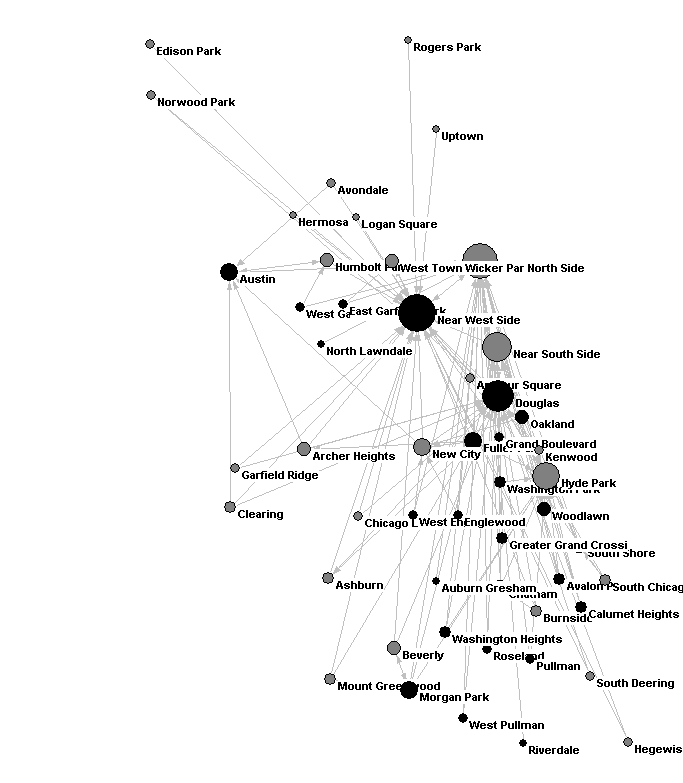


A B C

## *Appendix H: References*

Aral, Sinan and Marshall Van Alstyne. 2011. “The diversity-bandwidth trade-off.” *American Journal of Sociology*. 117(1):90–171.

Brashears, Matthew E., and Eric Quintane. 2018. “The Weakness of Tie Strength.” *Social Networks.* 55: 104-115.

Centola, Damon. 2021. *Change: How to make big change happen*. Little, Brown Spark.

Centola, Damon, and Michael Macy. 2007. “Complex Contagions and the Weakness of Long Ties.” *American Journal of Sociology.* 113: 702-34.

Graif, Corina, Alina Lungeanu, and Alyssa M. Yetter. 2017 "Neighborhood isolation in Chicago: Violent crime effects on structural isolation and homophily in inter-neighborhood commuting networks." *Social Networks.* 51: 40-59.

Hunter, David R. 2007. “Curved Exponential Family Models for Social Networks.” *Social Networks, Special section: Advances in exponential random graph (p*) models*. 29 (2): 216–30.

Larson, Jennifer M. 2017. “The weakness of weak ties for novel information diffusion.” *Applied Network Science.* 2(1):14.

Leifeld, Philip, Skyler J. Cranmer, and Bruce A. Desmarais. 2017. “xergm*: Extensions of Exponential Random Graph Models*.” R package version 1.8.2.

Levy, Michael A. 2016. “gwdegree: Improving interpretation of geometrically-weighted degree estimates in exponential random graph models.” *Journal of Open Source Software*. 1(3): 36.

McMillan, Cassie. 2019. “Tied Together: Adolescent Friendship Networks, Immigrant Status, and Health Outcomes.” *Demography.* 56:1075–1103.

Murray, Stephen, Joseph Rankin, and Dennis Magill. 1981. “Strong Ties and Job Information.” *Sociology of Work and Occupations.* 8: 119-36.

Neal, Zachary P. 2022. “Backbone: An R Package to Extract Network Backbones.” *PLoS ONE* 17 (5 May): 1–24.

Robins, Garry, Pip Pattison, Yuval Kalish, and Dean Lusher. 2007. “An introduction to exponential random graph (*p**) models for social networks.” *Social Networks.* 29: 173–191.

Serrano, M. Ángeles, Marián Boguñá, and Alessandro Vespignani. 2009. “Extracting the Multiscale Backbone of Complex Weighted Networks.” *Proceedings of the National Academy of Sciences of the United States of America* 106 (16): 6483–88.

Snijders, Tom A. B., Philippa E. Pattison, Garry L. Robins, and Mark S. Handcock. 2006. “New Specifications for Exponential Random Graph Models.” *Sociological Methodology.* 36 (1): 99–153.

Wasserman, Stanley, and Katherine Faust. 2010. *Social Network Analysis: Methods and Applications, Structural Analysis in the Social Sciences*, Cambridge University Press, Cambridge.
